# Mechanistic Exploration of *Carpobrotus edulis* Metabolites in Type 2 Diabetes Intervention Through Integrated Computational Approaches

**DOI:** 10.1155/bmri/9170020

**Published:** 2025-09-24

**Authors:** Halimat Yusuf Lukman, Athika Rampadarath, Stephen Amoo, Saheed Sabiu

**Affiliations:** ^1^ Department of Biotechnology and Food Science, Durban University of Technology, Durban, South Africa, dut.ac.za; ^2^ Agricultural Research Council-Vegetables, Industrial and Medicinal Plants, Pretoria, South Africa; ^3^ Unit for Environmental Science and Management, North-West University, Potchefstroom, South Africa, nwu.ac.za

**Keywords:** *Carpobrotus edulis*, molecular dynamics simulation, network pharmacology, signaling pathway, Type 2 diabetes mellitus

## Abstract

Despite the reported antidiabetic potential of *Carpobrotus edulis*, there is still a dearth of information on its modulatory role on the genes and signaling pathways implicated in Type 2 diabetes mellitus (T2DM). This study evaluated the gene–compound–pathways to lend scientific credence to the antidiabetic molecular mechanism of action of *C. edulis* using network pharmacology method. The results revealed that 11 metabolites of *C. edulis* that displayed oral drug‐likeness properties presented a network of 34 common genes with T2DM. While the gene ontology analysis revealed negative regulation of apoptotic, plasma membrane, and protein kinase as the biological parameters involved, the Kyoto Encyclopedia of Genes and Genomes analysis identified endocrine resistance (ER) signaling pathway as the most significant functional parameter. The ER signaling pathway had estrogen receptors 1 and 2 (ESR1 and ESR2) as the most enriched genes implicated in T2DM relative to *C. edulis*. Interestingly, the top ranked *C. edulis* metabolites displayed higher binding affinities (ranging from −39.98 to −49.67 kcal/mol for ESR1 and −33.21 to −58.59 kcal/mol for ESR2) than the standard drugs (metformin and tamoxifen [−14.21 and −12.34 kcal/mol for ESR1 and −20.65 and −47.92 kcal/mol for ESR2, respectively]), with catechin (−49.67 kcal/mol) and epicatechin (−58.59 kcal/mol) specifically displaying the highest binding free energies for ESR1 and ESR2, respectively. The greater binding interactions, stability, and structural orientation exhibited by *C. edulis* metabolites further substantiated their modulatory role on the genes. Overall, the significant binding affinities, stabilities, and interactions observed with the top ranked *C. edulis* metabolites, especially catechin and epicatechin with the two hub genes, suggest that *C. edulis* possibly elicits antidiabetic activity via enhancement of cellular glucose uptake and insulin sensitivity. However, further preclinical and clinical studies on the potential of *C. edulis* metabolites as potential drug candidates against T2DM are encouraged.

## 1. Introduction

Network pharmacology (NP) is a computational tool that systematically elucidates the mechanism of action of bioactive compounds in the management and/or treatment of disorders using the multitarget and multicomponent approach [1, 2]. The technique has been shown to significantly enhance the success rate of clinical trials and modification of existing therapeutic drugs as it affords the development of drugs with improved efficacy, reduced adverse effects, reduced cost of production, enhanced drug‐target interaction, and possible regulation of signaling pathways [3, 4]. With NP, the underlying molecular mechanism of action of bioactive compounds in the treatment of diseases including diabetes has been explored [5–7]. For example, the antidiabetic mechanism of action of *Khaya grandifoliola* study of Akoonjee et al. [8] demonstrated that cholestane‐3,26‐diol‐22‐one and linolelaidic acid are the top metabolites that significantly interacted with PRKCA and MMP2, respectively, in the advanced glycation end products–receptor and advanced glycation end products signaling pathway [8], while Maradesha et al. [9] showed that caffeic acid, deoxysappanone B 7,3 ^′^‐dimethyl ether acetate, and syringic acid identified in the methanolic extract of whole green jackfruit flour had high binding affinity with MAPK3 of insulin signaling pathway [9].

The annual death record and projected increasing prevalence of diabetes [10, 11] coupled with the accompanying limitations with conventional interventions [12, 13] has aroused research interest in understanding the fundamental mechanism of action of antidiabetic plants toward the development of more effective, safer, and target‐specific antidiabetic drugs. Fortunately, medicinal plants have shown to be alternative source of drugs and composed of active metabolites that allow binding and modulation of cellular targets involved in the pathology of different diseases [14]. While several studies have employed NP to elucidate the molecular mechanism of medicinal plants and their metabolites against T2DM [15–20], there are no studies adopting NP for *Carpobrotus edulis* in diabetes management to date.

*C. edulis* (L.) N. E. Br. is a creepy mat‐forming, edible halophytic succulent plant widely distributed in different coastal areas, indigenous to South Africa, particularly in the Eastern and Western Cape regions [21–23]. The therapeutic effects of *C. edulis* have been widely reported and attributed to the presence of bioactive constituents such as gallic acid, ellagic acid, ferulic acid, rutin, luteolin‐7‐O‐glucoside, epicatechin, uvaol, and procyanidin [24, 25]. To establish the antidiabetic effect of *C. edulis*, Mulaudzi et al. [26] reported that the ferulic acid and ellagic acid of *C. edulis* probably accounted for the inhibition of protein glycation observed in the glycosylated bovine serum albumin model. Hafsa et al. [27] reported the alpha‐glucosidase inhibitory effect of *C. edulis* extracts. Additionally, Sabiu et al. [28] profiled the phenolic constituents of *C. edulis* and studied their antidiabetic effect using in vitro and molecular dynamics (MD) simulation approaches. The study concluded that the phenolic contents of *C. edulis* displayed in vitro enzymatic inhibitory potential, binding stability, and compactness with the investigated enzymes.

Despite the commendable reports on the antidiabetic activity of *C. edulis*, there is still a paucity of information on its molecular mechanism of action with a focus on gene–compound–target to date. Hence, this study investigated the molecular mechanism of action of *C. edulis* metabolites in T2DM intervention using integrated NP and MD simulation strategies.

## 2. Materials and Methods

### 2.1. Data Mining for *C. edulis* Metabolites

Twenty‐four *C. edulis* metabolites were obtained through data mining; 11 were retrieved from literature [28] and 13 from the PubMed database (https://pubmed.ncbi.nlm.nih.gov/?term=Carpobrotus%2520edulis%2520chemicals). The metabolites identified were screened for drug‐likeness properties using the Swiss ADME server (http://swissadme.ch/index.php) [29].

### 2.2. Acquisition and Preparation of *C. edulis* Metabolites and T2DM Targets

The target genes of *C. edulis* metabolites with oral drug‐likeness properties were obtained by inserting their Simplified Molecular Input Line Entry System (SMILES) from PubChem (https://pubchem.ncbi.nlm.nih.gov/) into the SEA (Similarity Ensemble Approach) (http://sea.bkslab.org/) and Swiss Target Prediction (STP) (http://www.swisstargetprediction.ch/) databases. The GeneCards (https://www.genecards.org) and DisGeNeT (https://www.disgenet.org/search) databases were used to obtain T2DM target genes [30]. The VENNY 2.1 tool (https://bioinfogp.cnb.csic.es/tools/venny/) was used to identify the overlapping (common) genes between T2DM and *C. edulis* metabolites.

### 2.3. Creation of Intersecting Target Network

The protein–protein interaction (PPI) (*Homo sapiens*) of the overlapping genes of T2DM and *C. edulis* metabolites was constructed by inserting the overlapping genes into the Search Tool for the Retrieval of Interacting Genes/Proteins (STRING) database (https://string-db.org/). A confidence level > 0.4 was used for protein queries. The CytoHubba module in Cytoscape (Cytoscape 10.0 software) [31] was used for the whole network classification. The Cytoscape’s merge algorithm plugin was used to build a protein–compound–target (PCT) graphical web by merging the identified most enriched pathway in the PPI and its target genes with *C. edulis* metabolites. The network topology parameters of the PCT were analyzed, and their interactions were shown by the nodes and edges. A greater impact is achieved when there are more interacting nodes [32]. The degree of interaction of the most enriched gene was determined according to Equation (1) [33]:

(1) DegV=NV,

where *V* is the each node’s neighbors and *N*(*v*) is a node’s neighbors.

### 2.4. Gene Ontology (GO) and Kyoto Encyclopedia of Genes and Genomes (KEGG) Analyses of the Overlapping Targets

The official gene symbol identifier for *Homo sapiens* of the Database for Annotation, Visualization, and Integrated Discovery (DAVID) database (https://david.ncifcrf.gov/tools.jsp) [15] was used to interpret the GO of the overlapping targets. The GO analysis shows biological parameters such as biological processes (BPs), molecular functions (MFs), and cellular components (CCs). The KEGG analysis, which shows the functionally enriched pathway of the overlapping targets, was analyzed using the STRING software [16]. The *Q* value was obtained by using a false discovery rate (FDR) of *p* value < 0.05. The bubble plot map for the KEGG and GO was visualized using the Weishengxin bioinformatics database (https://www.bioinformatics.com.cn/).

### 2.5. Molecular Docking and MD Simulation

The 3D structures of the most enriched genes ([estrogen receptor 1 [ESR1] [PDB ID:1X7R] and estrogen receptor 2 [ESR2] [PDB ID:1X7J]) identified in the most significant T2DM pathway were obtained from the RSCB Protein Data Bank (https://www.rcsb.org/). Their structures were optimized using UCSF Chimera software V1.14 [34]. The 3D structures of the most interacting *C. edulis* metabolites obtained from the PCT analysis and the standards (metformin and tamoxifen) were obtained from PubChem (https://pubchem.ncbi.nlm.nih.gov/). Subsequently, Gasteiger charges and nonpolar hydrogen (H) atoms were added to optimize the structures [35].

The optimized metabolites were subjected to docking using the Autodock Vina Plugin on Chimera into ESR1 and ESR2 binding pockets with a grid box defined with a space of 1 Å, centers (X: 16.595, Y: 31.9366, and Z: 12.4095) for ESR1 and (X: 31.3211, Y: 36.7317, and Z: 40.704) for ESR2 [36]. Docking validation to prevent pseudopositive binding conformations was ensured at an initial root mean square deviation (RMSD) measurement of 0.5 and < 0.5 Å for ESR1 and ESR2 for their respective docked ligands and cocrystallized ligands after superimposition (Figure 1). The Top 4 metabolites with the highest docking scores were saved in PDB format for MD simulation. The graphical processing unit (GPU) version of the AMBER 18 package of the Centre for High Performance Computing (CHPC), Cape Town, South Africa, was employed in the MD simulation analysis using the AMBER 18 force field (FF18SB variant) [37]. The postdynamics analysis of the RMSD, root mean square fluctuation (RMSF), solvent accessible surface area (SASA), radius of gyration (RoG), and number of H‐bonds were analyzed using the CPPTRAJ module of the AMBER 18 suite, and the raw data obtained were plotted and analyzed using Origin software V18 (Origin Lab Corporation, Northampton, MA, United States) [38]. Binding free energy calculations of the metabolites and targets were run for a 100 ns simulation period, and the MD simulation trajectories were analyzed using molecular mechanics with the generalized born surface area method [39]. The interaction plots of the metabolites with the targets were thereafter visualized and analyzed using Discovery Studio V21.1.0 [40].

Figure 1Superimposition of (a) ESR1 with the best docked metabolite “epicatechin” (purple) and tamoxifen (green) on native ligand “genistein” (yellow) and (b) ESR2 with the best docked metabolite “ellagic acid” (purple) and “tamoxifen: (green) on native ligand “genistein” (yellow).

**Figure:**
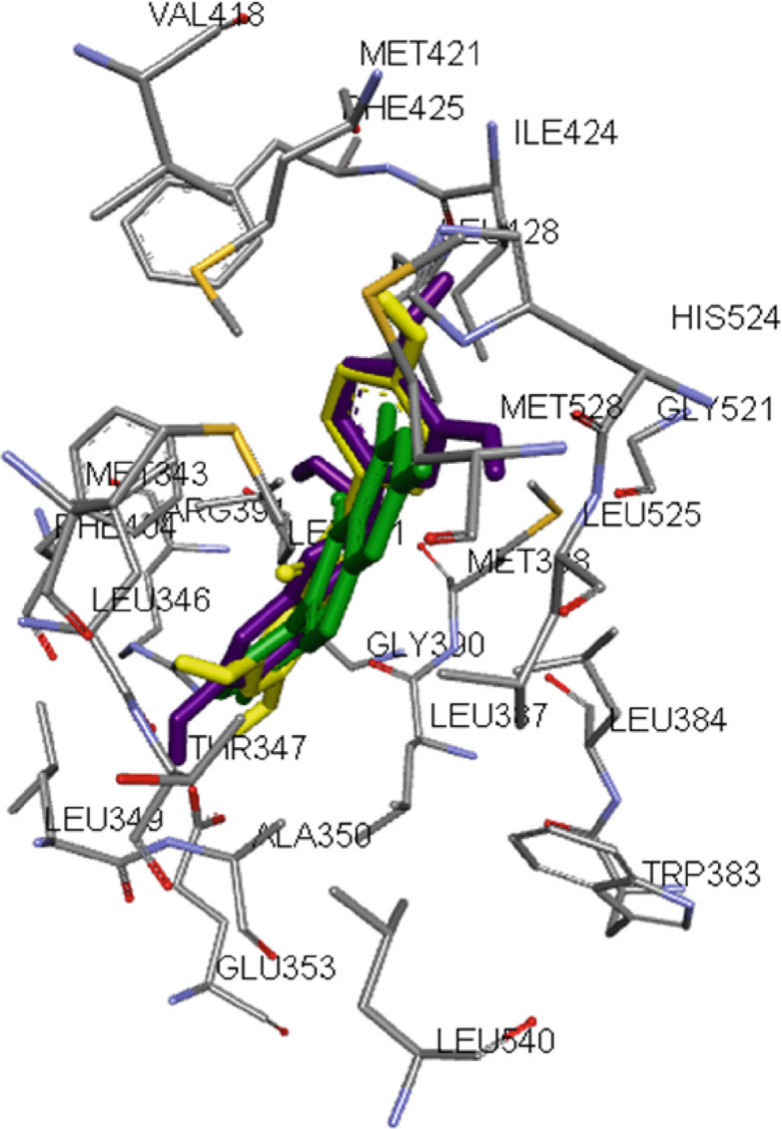
(a)

**Figure:**
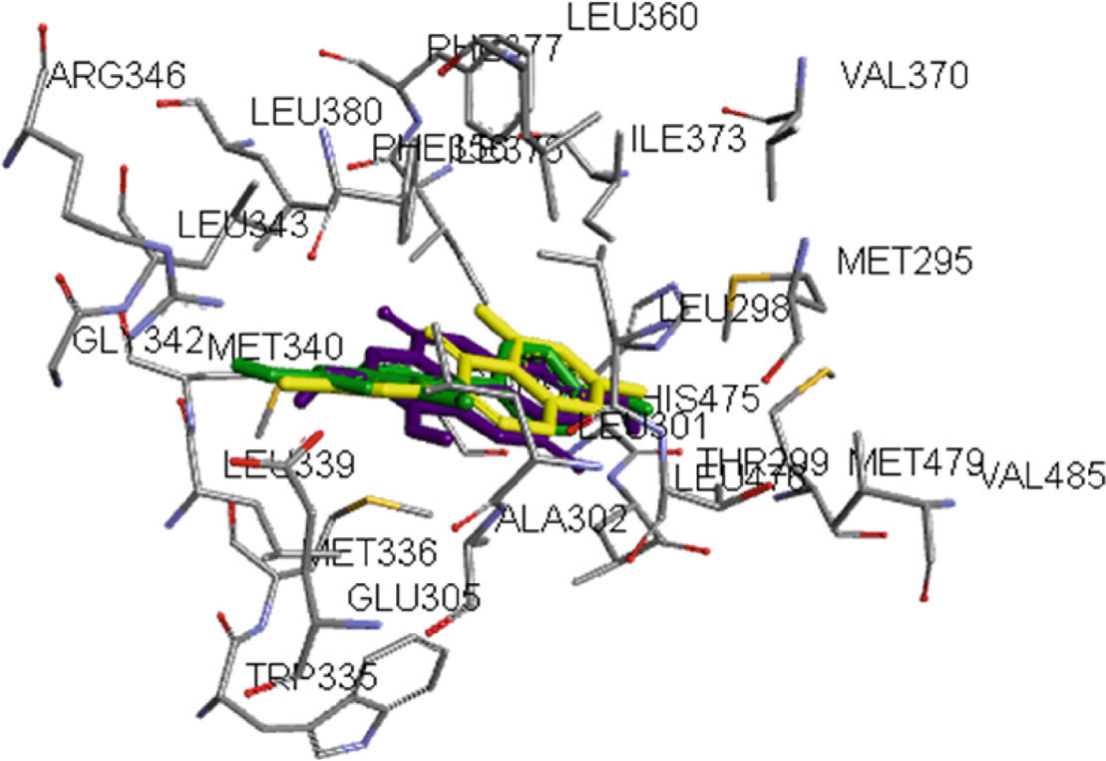
(b)

## 3. Results

### 3.1. Drug‐Likeness Filtering of *C. edulis* Metabolites

Of the 23 metabolites of *C. edulis* subjected to Lipinski’s filtering for drug‐likeness, only 11 passed the test (Table 1). The metabolites include caffeic acid, catechin, chlorogenic acid, ellagic acid, epicatechin, ferulic acid, gallic acid, myricetin, quinic acid, protocatechuic acid‐O‐glucoside, and sinapic acid (Table 1).

**Table 1:** Drug‐likeness properties of *C. edulis* metabolites.

**Metabolites**	**Pass Lipinski’s filter**	**Violations (V)**
1,3‐Dicaffeoylquinic acid	No	3 V: MW > 500, HBD > 10
Cacticin	No	2 V: HBD > 10, HBA > 5
Caffeic acid	Yes	—
Catechin	Yes	—
Catechin‐*O*‐glucoside	No	2 V: HBD > 10, HBA > 5
Chlorogenic acid	Yes	—
Ellagic acid	Yes	—
Epicatechin	Yes	—
Ferulic acid	Yes	—
Gallic acid	Yes	—
Hyperoside	No	2 V: HBD > 10, HBA > 5
Isoquercetin	No	2 V: HBD > 10, HBA > 5
Isorhamnetin‐3‐O‐rutinoside	No	3 V: MW > 500, HBD > 10, HBA > 5
Myricetin	Yes	—
Luteolin 7‐O‐beta‐D‐glucoside	No	2 V: HBD > 10, HBA > 5
Neohesperidin	No	3 V: MW > 500, HBD > 10, HBA > 5
Phloretin‐glucoside	No	HBD > 10, HBA > 5
Procyanidin	No	3 V: MW > 500, HBD > 10, HBA > 5
Protocatechuic acid‐*O*‐glucoside	Yes	—
Quinic acid	Yes	—
Rutin	No	3 V: MW > 500, HBD > 10, HBA > 5
Sinapic acid	Yes	—
Syringetin‐3‐O‐rutinoside	No	3 V: MW > 500, HBD > 10, HBA > 5

Abbreviations: HBA, number of hydrogen bond acceptor; HBD, number of hydrogen bond donor; MW, molecular weight.

### 3.2. Potential Overlapping Targets Between T2DM Targets and *C. edulis* Metabolites

A total of 59 (9.4%) common genes were observed between the STP (238) and SEA (449) genes for the 11 metabolites (Figure 2a). There were 2603 (18.7%) genes associated with T2DM overlapping between the 13,395 genes retrieved from GeneCards and 3134 genes retrieved from DisGeNet (Figure 2b). The overlapping genes obtained from the T2DM databases were examined for *C. edulis* metabolites–T2DM target genes relationship. Interestingly, 34 (1.3%) genes overlapped among the 59 genes associated with the metabolites and the 2603 genes associated with T2DM (Figure 2c).

Figure 2Common genes between (a) STP and SEA genes retrieved for *C. edulis*, (b) DisGeNet and GeneCards genes retrieved for T2DM, and (c) *C. edulis* metabolites and T2DM genes.

**Figure:**
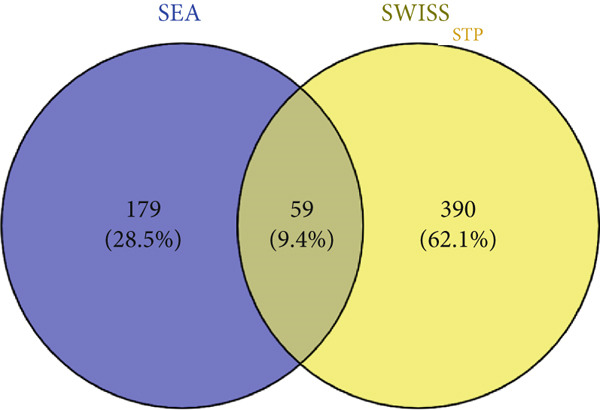
(a)

**Figure:**
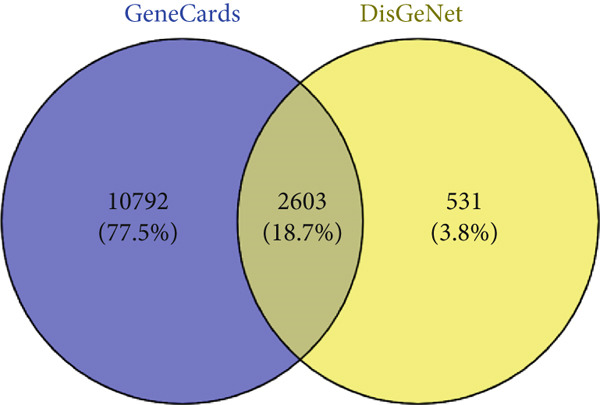
(b)

**Figure:**
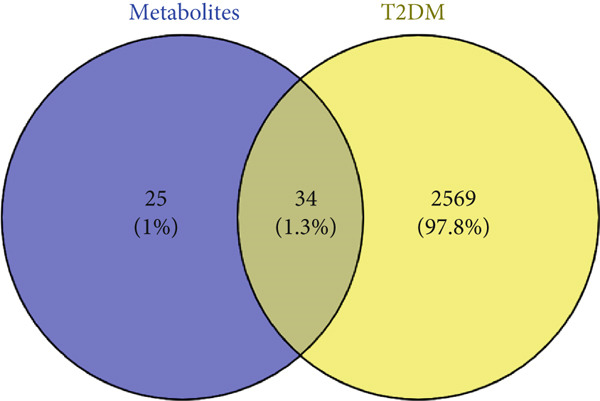
(c)

### 3.3. PPI of the Overlapping Genes in *C. edulis* Metabolites and T2DM

**Figure 3:**
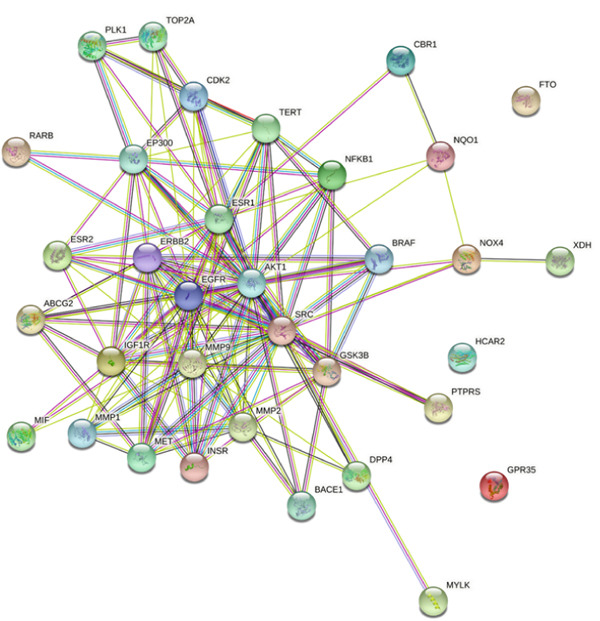
PPI of the 34 common genes of T2DM and *C. edulis* metabolites.

The PPI of the overlapping 34 genes associated with *C. edulis* metabolites is presented in Figure 3. The 34 genes associated with T2DM when introduced into the STRING database revealed a STRING algorithm of a network of 34 nodes connected by 141 edges. Three genes—FTO, HCAR2, and GPR35—were exclusive to the network (Figure 3).

### 3.4. GO and KEGG Pathway Enrichment Analyses of the 34 Overlapping Genes

**Figure 4:**
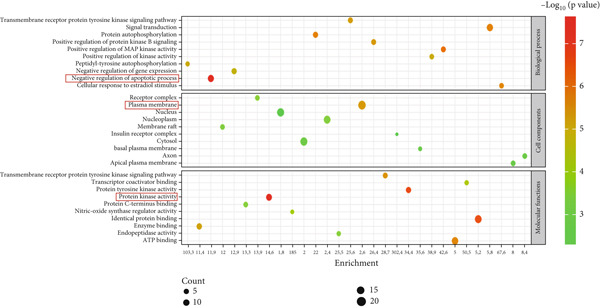
Bubble map of the top 10 GO annotations for the 34 common targets.

The Top 10 BP, MF, and CC of the 34 overlapping genes are shown in Figure 4. The negative regulation of the apoptotic process (*p* value = 1.1 × 10^−8^) was the most enriched BP out of the 146 pathways associated with the 34 genes common to *C. edulis* and T2DM. The Top 10 MF showed protein kinase activity (*p* value = 1.2 × 10^−8^) as the most enriched out of 46 MFs, while the Top 10 CC revealed the plasma membrane (*p* value = 2.4 × 10^−6^) as the most enriched out of 29 CCs associated with the 34 genes common to *C. edulis* and T2DM.

**Figure 5:**
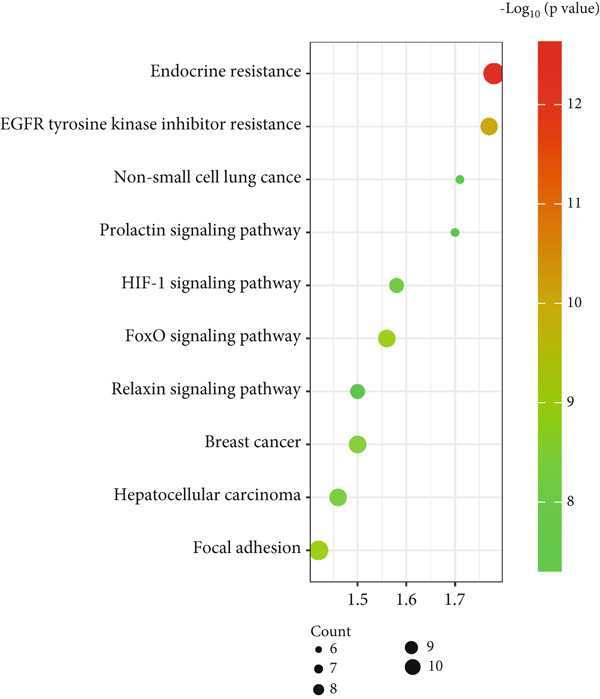
KEGG bubble map enrichment analysis of the Top 10 signaling pathways associated with the 34 common genes.

The KEGG pathway enrichment analysis for the Top 10 signaling pathways identified in the T2DM–*C. edulis* functional process is presented in Figure 5. Forty‐eight (48) signaling pathways associated with the 34 genes were identified at a threshold level of FDR at *p* value < 0.05 (Table 2). The most significantly enriched signaling pathway obtained was endocrine resistance. Other important signaling pathways observed were EGFR tyrosine kinase inhibitor resistance, focal adhesion, and Fox O, among others (Table 2).

**Table 2:** The signaling pathways and target genes related to *C. edulis* and T2DM.

**Signaling pathway**	**False discovery rate**	**Genes**
Endocrine resistance	2.48 × 10^−13^	MMP2, IGF1R, ERBB2, EGFR, BRAF, ESR2, MMP9, SRC, ESR1, and AKT1
EGFR tyrosine kinase inhibitor resistance	1.17 × 10^−10^	IGF1R, ERBB2, EGFR, BRAF, MET, GSK3B, SRC, and AKT1
Focal adhesion	2.14 × 10^−9^	IGF1R, ERBB2, EGFR, BRAF, MET, GSK3B, MYLK, SRC, and AKT1
Fox O	2.16 × 10^−9^	EP300, CDK2, IGF1R, EGFR, BRAF, PLK1, INSR, and AKT1
Breast cancer	5.36 × 10^−9^	IGF1R, ERBB2, EGFR, BRAF, GSK3B, ESR2, ESR1, and AKT1
Hepatocellular carcinoma	1.04 × 10^−9^	IGF1R, EGFR, BRAF, TERT, MET, NQO1, GSK3B, and AKT1
HIF‐1	2.15 × 10^−8^	NFKB1, EP300, IGF1R, ERBB2, EGFR, INSR, and AKT1
Prolactin	6.95 × 10^−8^	NFKB1, GSK3B, ESR2, SRC, ESR1, and AKT1
Relaxin	6.95 × 10^−8^	MMP2, NFKB1, EGFR, MMP1, MMP9, SRC, and AKT1
Non–small cell lung cancer	6.95 × 10^−8^	ERBB2, EGFR, BRAF, MET, RARB, and AKT1
Estrogen	7.38 × 10^−8^	MMP2, EGFR, ESR2, MMP9, SRC, ESR1, and AKT1
PI3K‐Akt	1.37 × 10^−7^	NFKB1, CDK2, IGF1R, ERBB2, EGFR, INSR, MET, GSK3B, and AKT1
ErbB	1.59 × 10^−7^	ERBB2, EGFR, BRAF, GSK3B, SRC, and AKT1
MAPK	5.09 × 10^−7^	NFKB1, IGF1R, ERBB2, EGFR, BRAF, INSR, MET, and AKT1
Rap1	9.12 × 10^−7^	IGF1R, EGFR, BRAF, INSR, MET, SRC, and AKT1
Fluid shear stress and atherosclerosis	1.60 × 10^−6^	MMP2, NFKB1, NQO1, MMP9, SRC, and AKT1
Pancreatic cancer	2.88 × 10^−6^	NFKB1, ERBB2, EGFR, BRAF, and AKT1
Thyroid hormone	2.43	EP300, GSK3B, SRC, ESR1, and AKT1
Alzheimer disease	2.54 × 10^−5^	NFKB1, NOX4, BRAF, INSR, BACE1, GSK3B, and AKT1
Ras	2.68 × 10^−5^	NFKB1, IGF1R, EGFR, INSR, MET, and AKT1
Renal cell carcinoma	6.30e × 10^−5^	EP300, BRAF, MET, and AKT1
mTOR	6.48 × 10^−5^	IGF1R, BRAF, INSR, GSK3B, and AKT1
Chemokine	1.5 × 10^−4^	NFKB1, BRAF, GSK3B, SRC, and AKT1
IL‐17	1.7 × 10^−4^	NFKB1, MMP1, GSK3B, and MMP9
AGE‐RAGE in diabetic complications	2.1 × 10^−4^	MMP2, NFKB1, NOX4, and AKT1
cAMP	2.3 × 10^−4^	NFKB1, EP300, BRAF, HCAR2, and AKT1
Insulin resistance	2.7 × 10^−4^	NFKB1, INSR, GSK3B, and AKT1
Insulin	5.6 × 10^−4^	BRAF, INSR, GSK3B, and AKT1
Nonalcoholic fatty liver disease	8.1 × 10^−4^	NFKB1, INSR, GSK3B, and AKT1
B cell receptor	1.9 × 10^−3^	NFKB1, GSK3B, and AKT1
GnRH	2.7 × 10^−3^	MMP2, EGFR, and SRC
C‐type lectin receptor	3.8 × 10^−3^	NFKB1, SRC, and AKT1
T cell receptor	3.8 × 10^−3^	NFKB1, GSK3B, and AKT1
TNF	4.9 × 10^−3^	NFKB1, MMP9, and AKT1
Growth hormone synthesis, secretion, and action	5.6 × 10^−3^	EP300, GSK3B, and AKT1
AMPK	5.8 × 10^−3^	IGF1R, INSR, and AKT1
Phospholipase D	9.5 × 10^−3^	EGFR, INSR, and AKT1
Oxytocin	9.8 × 10^−3^	EGFR, MYLK, and SRC
JAK‐STAT	1.16 × 10^−2^	EP300, EGFR, and AKT1
cGMP‐PKG	1.19 × 10^−2^	INSR, MYLK, and AKT1
Axon guidance	1.47 × 10^−2^	MET, GSK3B, and SRC
Regulation of lipolysis in adipocytes	1.66 × 10^−2^	INSR and AKT1
VEGF	1.81 × 10^−2^	SRC and AKT1
Long‐term depression	1.87 × 10^−2^	IGF1R and BRAF
Adipocytokine	2.43 × 10^−2^	NFKB1 and AKT1
Glucagon	4.62 × 10^−2^	EP300 and AKT1
Parathyroid hormone synthesis, secretion, and action	4.70 × 10^−2^	EGFR and BRAF

### 3.5. Network Interaction of Endocrine Resistance Pathway, Genes, and *C. edulis* Metabolites

The PCT interaction of the most enriched pathway, its genes, and *C. edulis* metabolites showed a network of 48 edges and 22 nodes. The nodes comprised one signaling pathway, 10 genes, and 11 metabolites (Figure 6a). The gene–gene interaction of the 10 genes (MMP2, IGF1R, ERBB2, EGFR, BRAF, ESR2, MMP9, SRC, ESR1, and AKT1) associated with the endocrine resistance pathway is shown in Figure 6b. A network was constructed for the endocrine resistance pathway enriched genes with the *C. edulis* metabolites (Figure 7 and Figure S1). The ESR1 and ESR2 were the most interacting genes. ESR1 interacted with seven metabolites (Figure 7a), while ESR2 interacted with eight metabolites (Figure 7b). The other genes interacted with fewer metabolites of *C. edulis* (Figure S1).

Figure 6(a) PCT network of *C. edulis* metabolites and T2DM enriched pathway and targets. (b) Interaction of endocrine resistance pathway genes.

**Figure:**
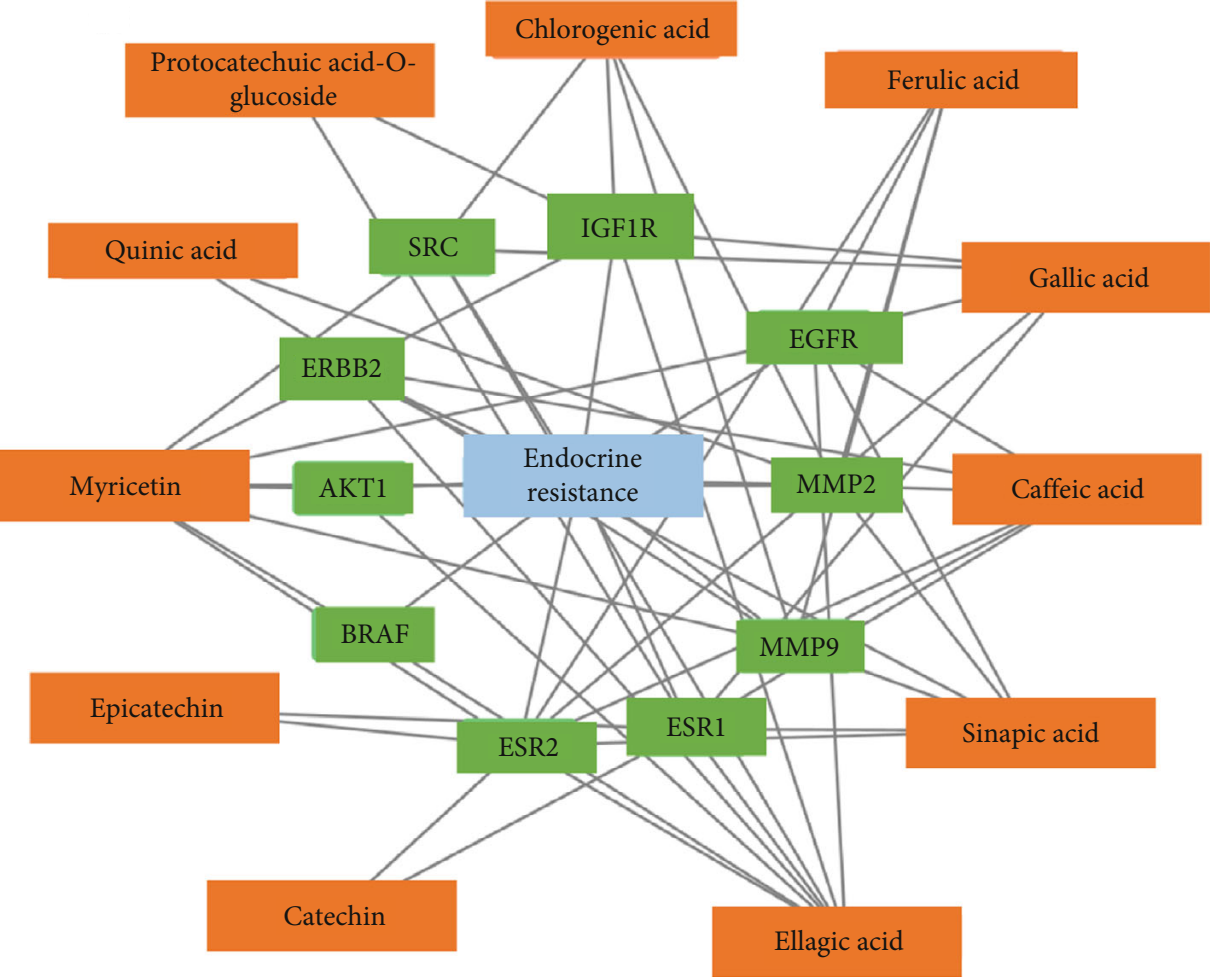
(a)

**Figure:**
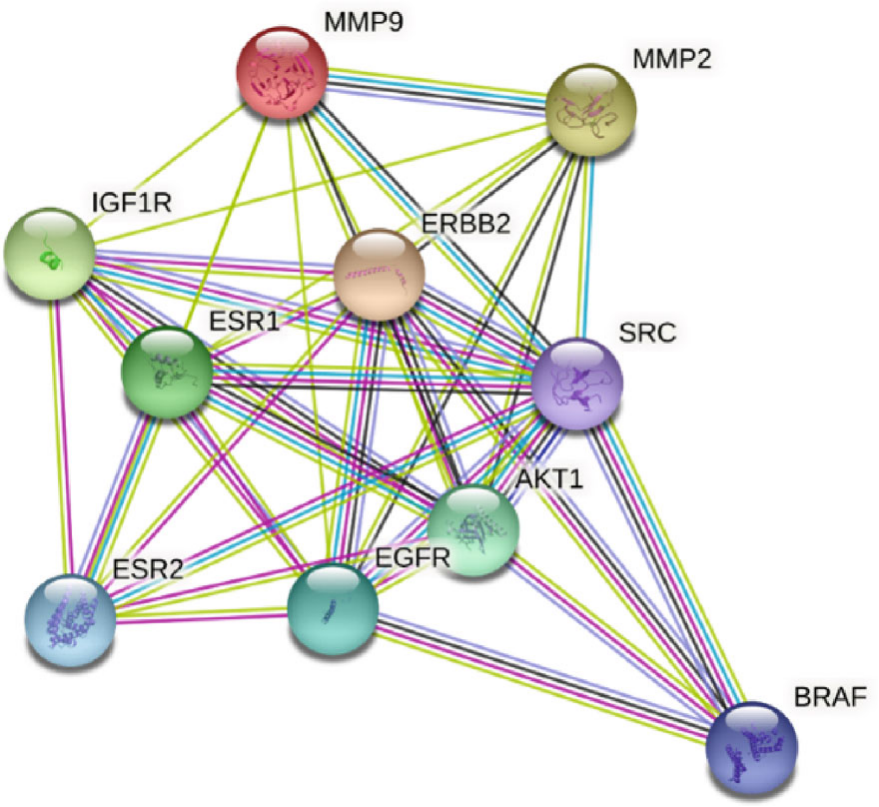
(b)

Figure 7Network construction of *C. edulis* metabolites (yellow) with genes (yellow) related to the endocrine resistance signaling pathway (yellow): (a) seven metabolites associated with ESR1 and (b) eight metabolites associated with ESR2.

**Figure:**
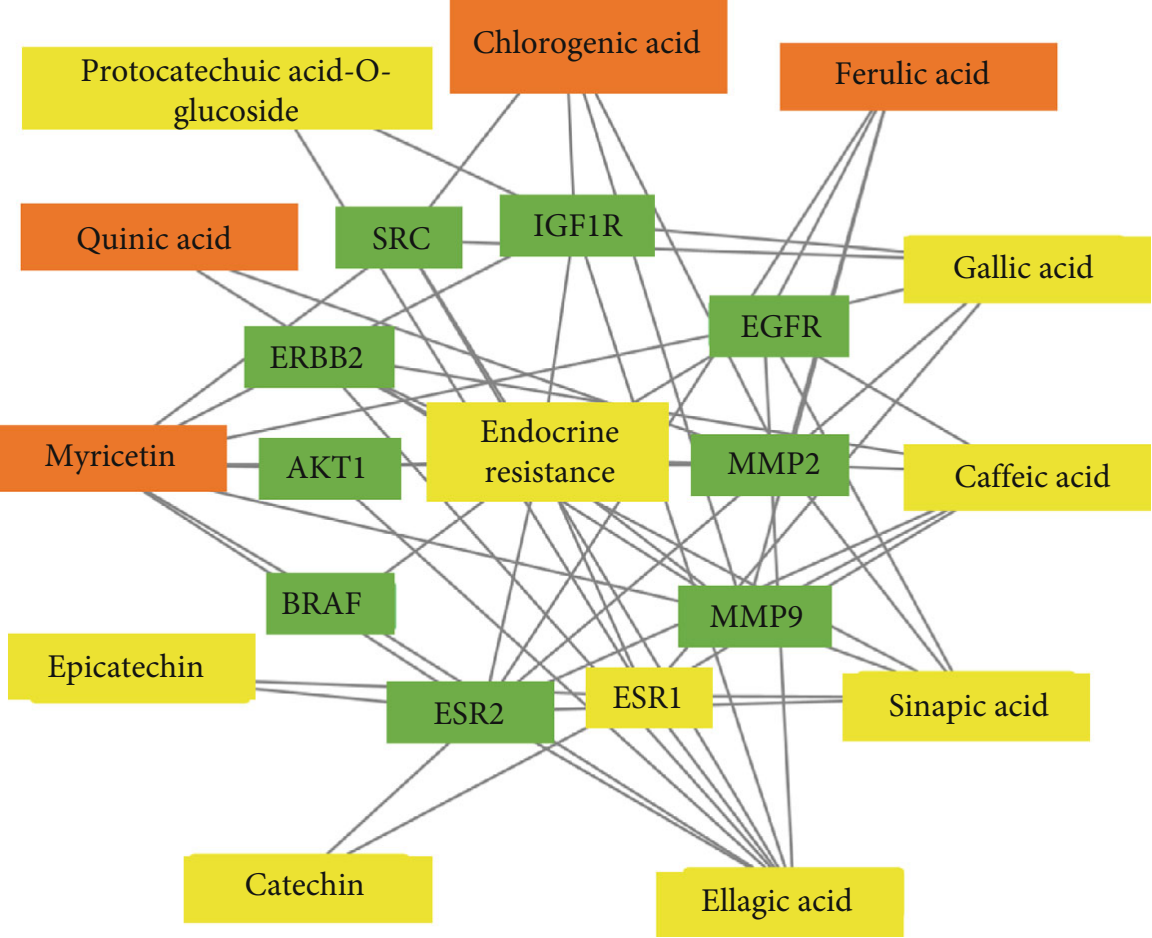
(a)

**Figure:**
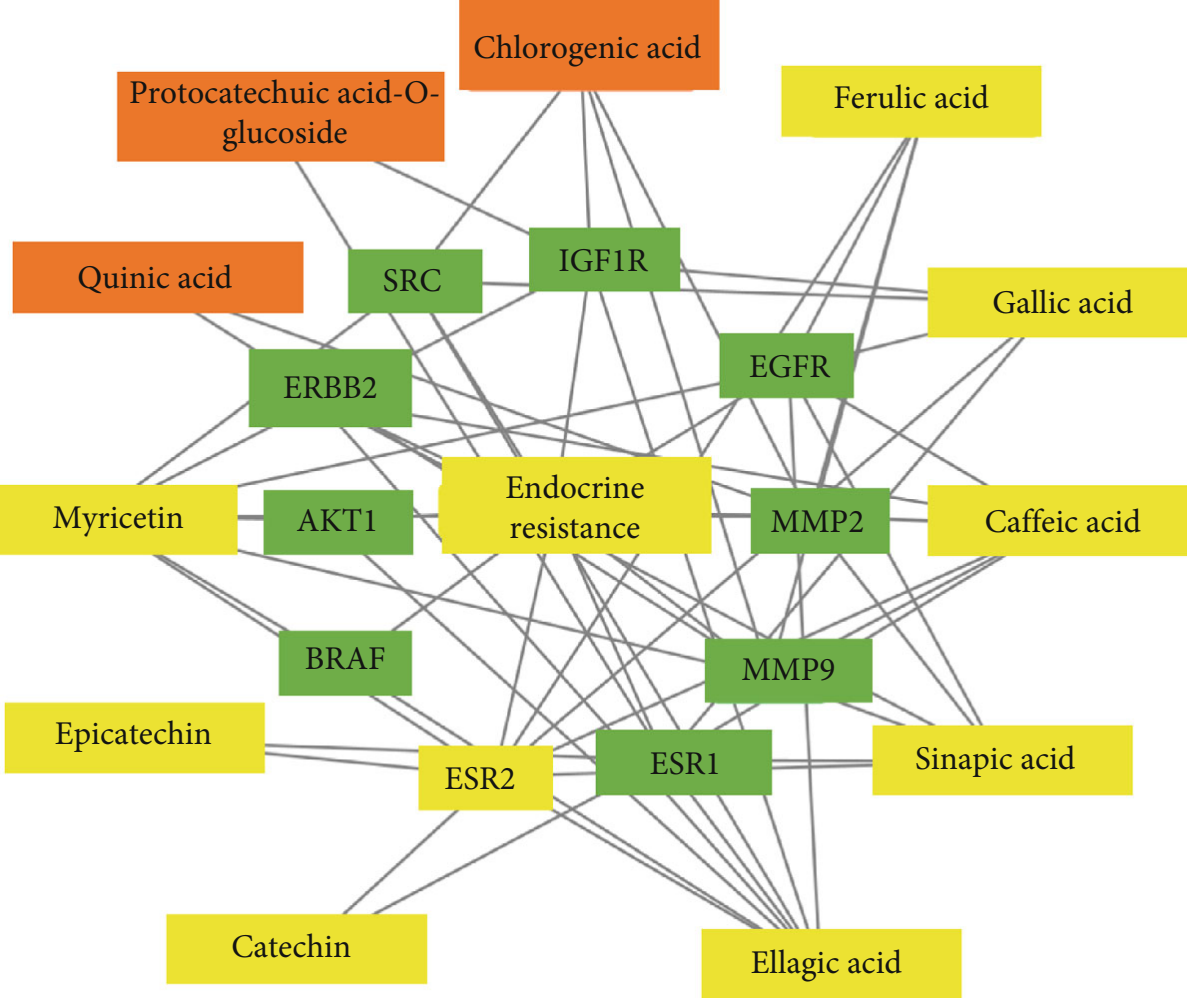
(b)

### 3.6. Docking Interaction of *C. edulis* Metabolites With Endocrine Resistance Signaling Pathway Enriched Genes

The docking scores and 2D interaction of *C. edulis* metabolites with ESR1 and ESR2 are displayed in Table S1 and Figures 2 and 3. However, the Top 4 metabolites with the highest docking scores and their energy component parameters are presented in Table 3. Ellagic acid had the highest negative docking score (−9.4 kcal/mol) but showed a lower binding free energy (−32.97 ± 3.58 kcal/mol) than catechin and epicatechin, which had −9.1 and −8.7 docking scores, respectively (Table 3). Metformin had the least docking score and binding free energy (−5.1 and −14.21 ± 4.11 kcal/mol, respectively) with ESR1, while tamoxifen presented a high positive binding free energy (12.39 ± 6.02 kcal/mol). For ESR2, *C. edulis* metabolites had higher negative docking scores and binding free energy than the standards (Table 3). Epicatechin had both the highest docking score and binding free energy of −8.8 and −58.59 ± 3.66 kcal/mol, respectively, followed by ellagic acid and catechin with the same docking scores (−8.5 kcal/mol) but different binding free energies (Table 3).

**Table 3:** Docking scores and energy components of Top 4 metabolites of *C. edulis* against ESR1 and ESR2 genes of T2DM.

**Complexes**	**kcal/mol**					
	**Docking score**	**Δ** **E**_**v** **d** **W**_	**Δ** **E**_**e** **l** **e** **c**_	**Δ** **G**_**g** **a** **s**_	**Δ** **G**_**s** **o** **l** **v**_	**Δ** **G**_**b** **i** **n** **d**_
*ESR1*						
Catechin	−9.1	−34.09 ± 3.48	−55.80 ± 5.57	−89.89 ± 4.90	40.22 ± 3.53	−49.67 ± 3.03
Ellagic acid	−9.4	−38.02 ± 2.67	−19.40 ± 6.72	−57.41 ± 7.12	24.44 ± 2.20	−32.97 ± 3.58
Epicatechin	−8.7	−37.41 ± 3.08	−40.91 ± 6.65	−78.33 ± 6.36	35.36 ± 3.21	−42.97 ± 4.00
Protocatechuic acid 3‐glucoside	−7.5	−41.07 ± 2.85	−26.59 ± 5.84	−67.66 ± 5.12	27.68 ± 3.88	−39.98 ± 3.58
Metformin	−5.1	−18.58 ± 2.30	−162.42 ± 17.21	−181.00 ± 17.53	166.29 ± 15.21	−14.21 ± 4.11
Tamoxifen	−6.8	−16.85 ± 7.45	−78.43 ± 20.50	−95.23 ± 23.24	82.89 ± 20.67	−12.34 ± 6.02
*ESR2*						
Catechin	−8.5	−36.67 ± 3.58	−47.07 ± 9.19	−83.75 ± 7.82	31.33 ± 2.75	−52.42 ± 5.82
Ellagic acid	−8.5	−23.35 ± 3.65	−46.68 ± 9.8	−70.04 ± 10.19	36.83 ± 5.02	−33.21 ± 5.74
Epicatechin	−8.8	−35.35 ± 3.53	−57.49 ± 6.83	−92.83 ± 5.67	38.24 ± 3.40	−58.59 ± 3.66
Myricetin	−8.4	−37.77 ± 3.57	−51.75 ± 4.91	−89.52 ± 4.14	37.56 ± 2.10	−51.96 ± 3.19
Metformin	−5.1	−20.09 ± 2.48	−95.55 ± 13.70	−115.64 ± 13.51	94.99 ± 12.32	−20.65 ± 5.50
Tamoxifen	−5.9	−55.49 ± 2.95	−103.71 ± 15.05	−159.20 ± 14.70	111.28 ± 13.29	−47.92 ± 3.36

Abbreviations: *Δ* *E*_elec_, electrostatic energy; *Δ* *E*_gas_, gas‐phase free energy; *Δ* *E*_*V* *d* *W*_, Van der Waals energy; *Δ* *G*_bind_, total binding free energy; *Δ* *G*_*s* *o* *l* *v*_, solvation free energy.

### 3.7. Postdynamic Trajectories of Top 4 *C. edulis* Metabolites Against ESR1 and ESR2 Genes

The postdynamic trajectories of the Top 4 *C. edulis* metabolites against ESR1 and ESR2 genes are shown in Figures 8 and 9. In the RMSD plot of ESR1, all the systems equilibrated at 0 ns with little or no divergence throughout the simulation period. However, all the metabolites had lower mean RMSD values than the apo‐ESR1 (1.84 Å), metformin (2.09 Å), and tamoxifen (2.02 Å) (Table 4). All the metabolites showed no significant prominent peaks in the RMSF plot and again had lower mean RMSF values compared to apo‐ESR1 (1.27 Å), metformin (1.27 Å), and tamoxifen (1.33 Å) (Table 4). In the RoG plot, the tamoxifen–ESR1 complex showed greater fluctuation, whereas other bound systems showed a relatively stable complex with epicatechin fluctuating the least, with a mean RoG value of 18.04, while protocatechuic acid 3‐glucoside (18.29 Å) had the highest value relative to apo‐ESR1 (18.20 Å) (Table 4). A stable SASA plot was observed over the simulation period. However, the standards had higher mean values than the metabolites. Catechin and epicatechin had lower SASA values than ellagic acid (Table 4). The H‐bond plot was stable for the complexes with little variation; however, catechin had slightly higher H‐bond number (Table 4).

Figure 8Comparative postdynamic data showing (a) RMSD, (b) RMSF, (c) RoG, (d) SASA, and (e) number of intramolecular H‐bond plots of catechin, ellagic acid, epicatechin, and protocatechuic acid 3‐glucoside of *C. edulis*, metformin, and tamoxifen with ESR1.

**Figure:**
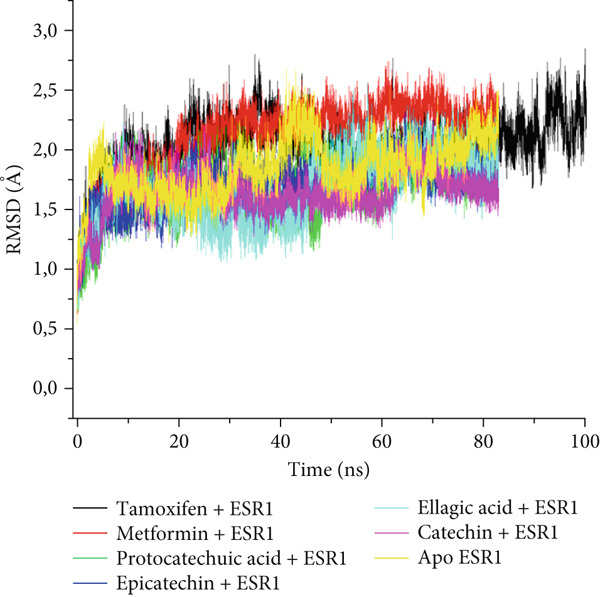
(a)

**Figure:**
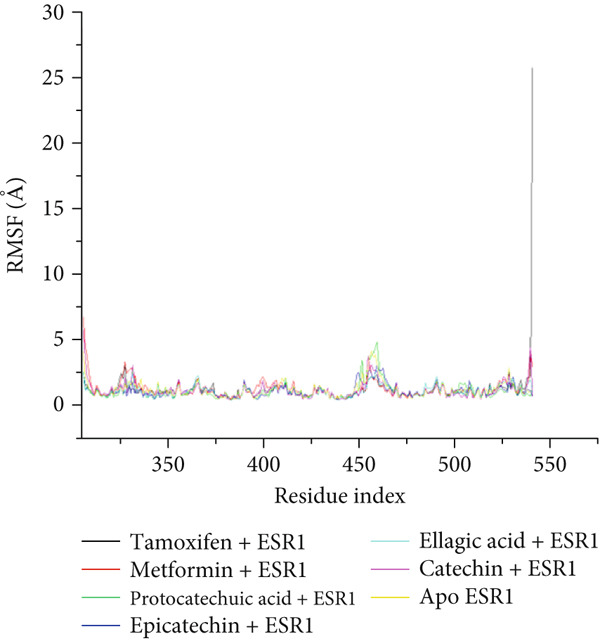
(b)

**Figure:**
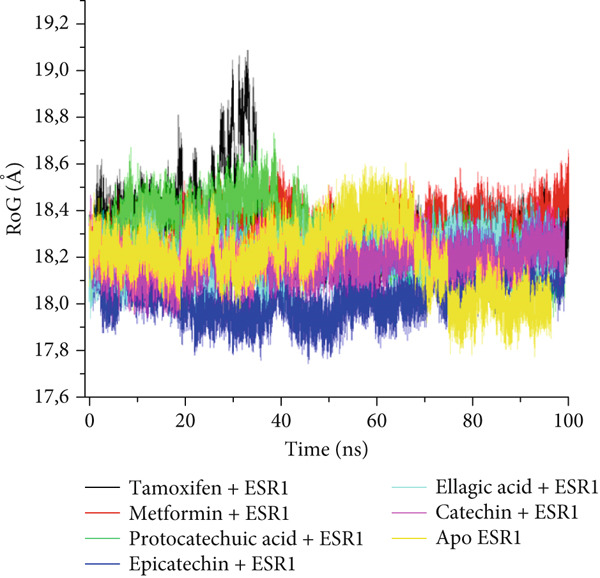
(c)

**Figure:**
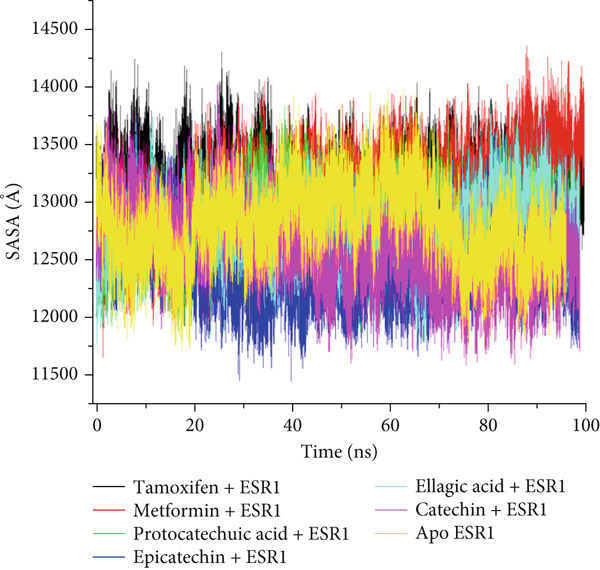
(d)

**Figure:**
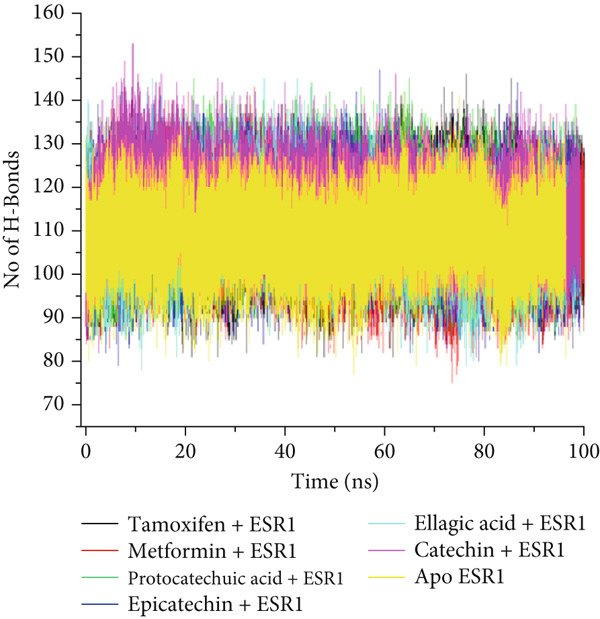
(e)

Figure 9Comparative postdynamic data showing (a) RMSD, (b) RMSF, (c) RoG, (d) SASA, and (e) number of intramolecular H‐bond plots of catechin, ellagic acid, epicatechin, and myricetin of *C.* edulis, metformin, and tamoxifen with ESR2.

**Figure:**
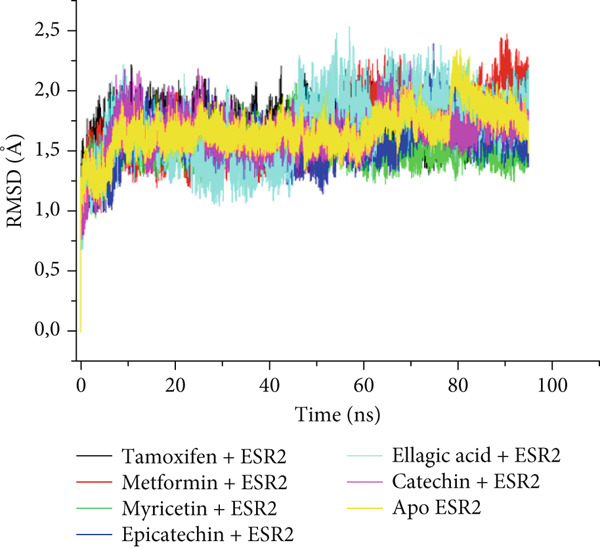
(a)

**Figure:**
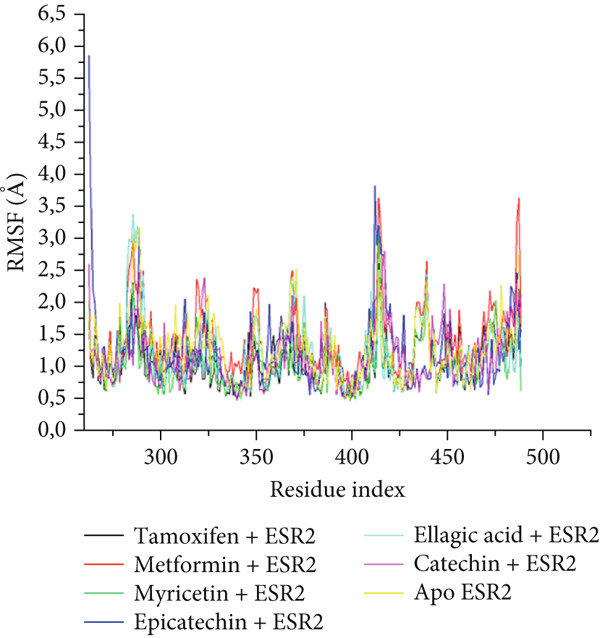
(b)

**Figure:**
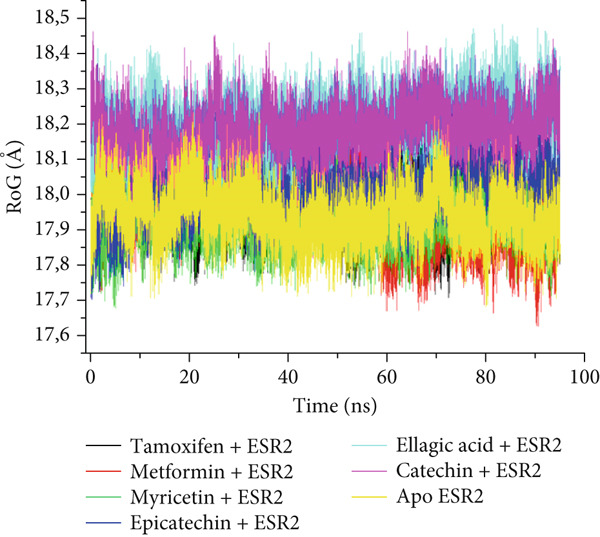
(c)

**Figure:**
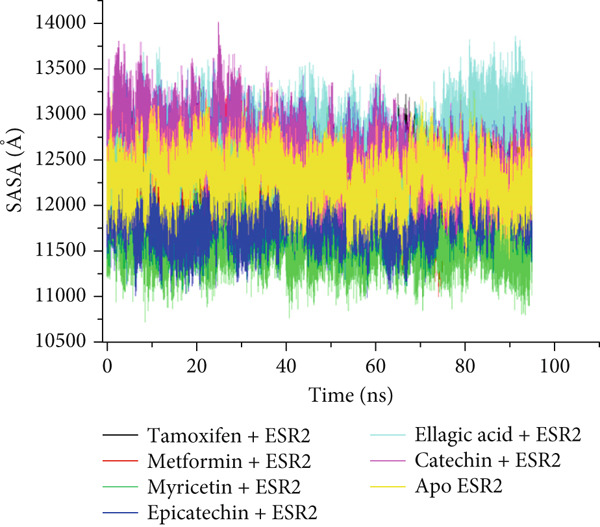
(d)

**Figure:**
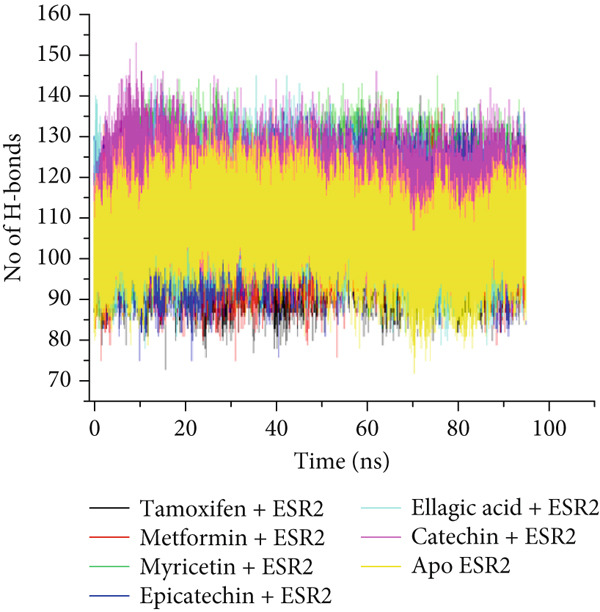
(e)

**Table 4:** Post‐MD simulation and hydrogen bond parameters of Top 4 metabolites of *C. edulis* following binding to ESR1 and ESR2 genes.

**Complexes**	**RMSD (Å)**	**RMSF (Å)**	**RoG (Å)**	**SASA (Å)**	**Number of H-bonds**
*ESR1*					
Apo‐ESR1	1.84 ± 0.23	1.27 ± 0.62	18.20 ± 0.14	12803.75 ± 310.24	108.72 ± 7.31
Catechin	1.63 ± 0.19	1.24 ± 0.65	18.18 ± 0.07	12589.51 ± 261.55	115.07 ± 7.57
Ellagic acid	1.66 ± 0.27	1.18 ± 0.45	18.21 ± 0.07	12752.51 ± 216.55	111.21 ± 7.82
Epicatechin	1.72 ± 0.19	1.20 ± 0.50	18.04 ± 0.09	12495.50 ± 287.17	110.90 ± 7.21
Protocatechuic acid 3‐glucoside	1.66 ± 0.23	1.20 ± 0.66	18.29 ± 0.11	12379.48 ± 252.95	114.16 ± 7.27
Metformin	2.09 ± 0.31	1.35 ± 0.72	18.31 ± 0.08	13079.40 ± 341.62	109.38 ± 7.65
Tamoxifen	2.02 ± 0.23	1.33 ± 1.66	18.31 ± 0.15	13096.03 ± 275.66	111.17 ± 7.61
*ESR2*					
Apo‐ESR2	1.68 ± 0.17	1.28 ± 0.5	17.95 ± 0.07	12298.32 ± 244.32	106.62 ± 7.73
Catechin	1.65 ± 0.18	1.22 ± 0.62	18.18 ± 0.07	12594.75 ± 325.12	115.23 ± 7.56
Ellagic acid	1.69 ± 0.26	1.16 ± 0.45	18.21 ± 0.07	12748.65 ± 262.79	111.26 ± 7.86
Epicatechin	1.58 ± 0.18	1.20 ± 0.52	18.03 ± 0.07	11908.18 ± 243.28	108.30 ± 7.47
Myricetin	1.53 ± 0.12	1.06 ± 0.40	17.94 ± 0.06	11609.05 ± 211.58	114.80 ± 7.51
Metformin	1.71 ± 0.25	1.40 ± 0.54	17.97 ± 0.09	12191.40 ± 300.38	107.43 ± 7.38
Tamoxifen	1.69 ± O.12	1.07 ± 0.40	17.98 ± 0.07	12246.60 ± 248.65	104.74 ± 7.17

Abbreviations: RMSD, root mean square deviation; RMSF, root mean square fluctuation; RoG, radius of gyration; SASA, solvent accessible surface area.

For ESR2, all the systems equilibrated at the 0 ns and were stable throughout the 100 ns simulation (Figure 9a). However, ellagic acid had a lower fluctuation around 20 and 45 ns, with a mean RMSD value of 1.69 Å, while myricetin had the lowest (1.53 Å) value (Table 4). The RMSF plot showed peaks between 275–300 and 410–425 amino acid residues (Figure 9b). Metformin (1.40 Å) displayed the highest mean RMSF value, while among the metabolites, myricetin (1.06 Å) had the least value and catechin had the highest (1.22 Å) (Table 4). In the RoG, the metabolites initially equilibrated; however, the metabolites showed slight deviation from the apo‐gene, except myricetin (Figure 9c). The mean RoG of myricetin (17.97 Å) was almost the same as the apo‐gene (17.98 Å). However, ellagic acid (18.21 Å) displayed the highest mean RoG among the metabolites in the bound system (Table 4). The SASA plot showed a converging complex, with ellagic acid and catechin deviating farther from the apo‐gene and myricetin closer to the apo‐ESR2 (Figure 9d). This trend is observed in their mean SASA values, where myricetin had 11609.05 Å while ellagic acid had 12748.65 Å (Table 4). A converging and stable number of H‐bond plots was observed in the ESR2–*C. edulis* metabolite complexes (Figure 9e). Interestingly, more H‐bond numbers were recorded in the metabolites than in the apo‐ESR2 and standards (Table 4).

### 3.8. Post‐MD Simulation Interaction Plots of the Top 4 Metabolites of *C. edulis* Following Binding to ESR1 and ESR2 Genes

The 2D interaction plots of *C. edulis* metabolites with ESR1 and ESR2 post 100 ns MD simulation are displayed in Figures 10 and 11, respectively. The metabolites interacted with several H (hydrophilic) and hydrophobic bonds and amino acid residues with the genes. For ESR1, throughout the simulation period, the catechin–ESR1 complex interacted with 19 bonds with one (Gly521) H‐bond at 0 ns. However, the complex maintained the same H‐bond number of two (Glu41 and Gly209) at the 50 and 100 ns simulation period (Figure 10a). Ellagic acid interacted with 20 bonds at 0 ns with 1 H‐bond (His 524) while 16 interaction bonds with three H‐bonds (Met76, Hie212, and Gly209) were observed at 100 ns (Figure 10b). In the epicatechin‐ESR1 complex, a stable number of 18 bonds interacted throughout the simulation period with the same number of H‐bonds (three); at 0 ns, His524, Gly521, and Glu353 were observed, while at 100 ns, Hie212, Glu41, and Gly209 existed (Figure 10c). A total of 20 bonds including three H‐bonds (Arg394, Gly521, and Leu346) existed between protocatechuic acid‐3‐glucoside and ESR1 at 0 ns, while 19 interacting bonds with four H‐bonds (Gly209, Hie212, Leu75, and Glu41) were formed at the end of the simulation period (Figure 10d). Metformin formed nine bonds (two H‐bonds: Leu346 and 387) and 10 bonds (two H‐bonds: Phe92 and Leu91) with ESR2 at 0 and 100 ns, respectively (Figure 10e), while tamoxifen formed 10 bonds (one H‐bond: Leu489) at 0 ns and nine bonds with no H‐bond interaction at the end of the simulation (Figure 10f).

Figure 102D postdynamic interaction of ESR2 with (a) catechin, (b) ellagic acid, (c) epicatechin, (d) myricetin, (e) metformin, and (f) tamoxifen at 0, 50, and 100 ns simulation.

**Figure:**
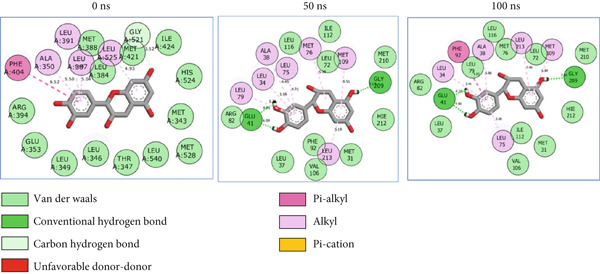
(a)

**Figure:**
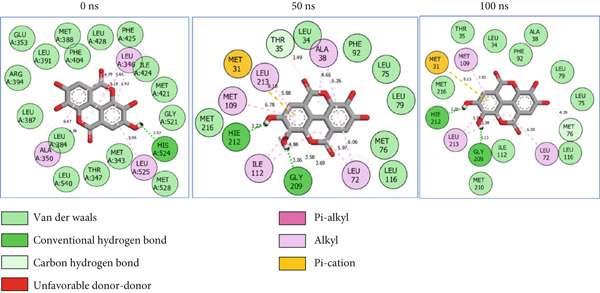
(b)

**Figure:**
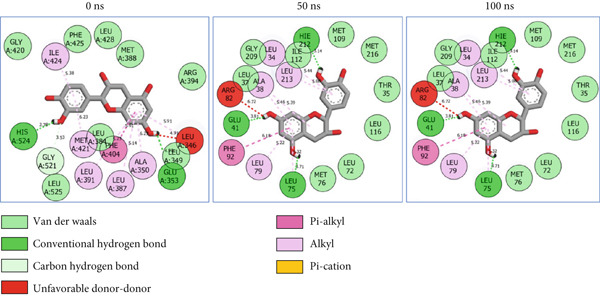
(c)

**Figure:**
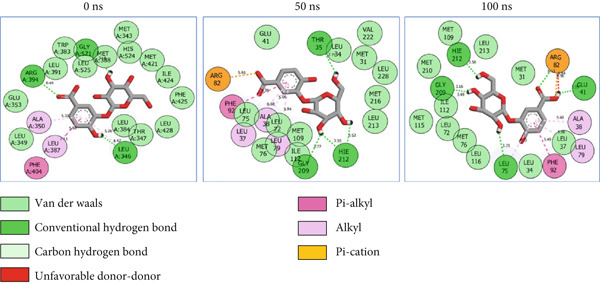
(d)

**Figure:**
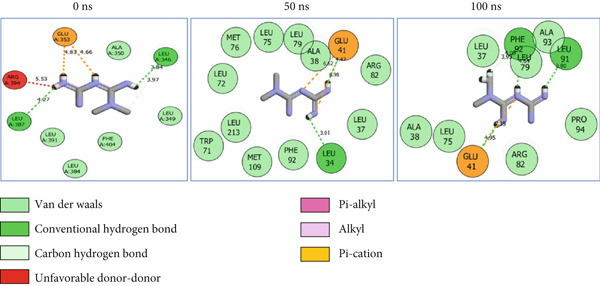
(e)

**Figure:**
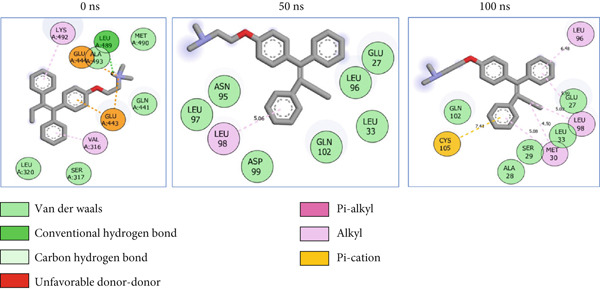
(f)

Figure 112D postdynamic interaction of ESR2 with (a) catechin, (b) ellagic acid, (c) epicatechin, (d) myricetin, (e) metformin, and (f) tamoxifen at 0, 50, and 100 ns simulation.

**Figure:**
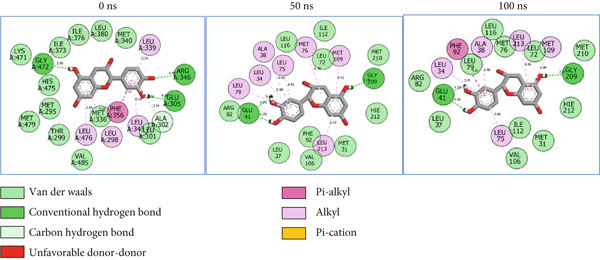
(a)

**Figure:**
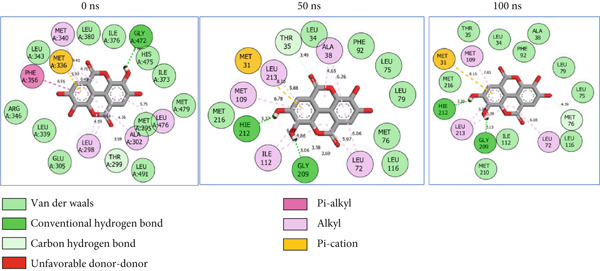
(b)

**Figure:**
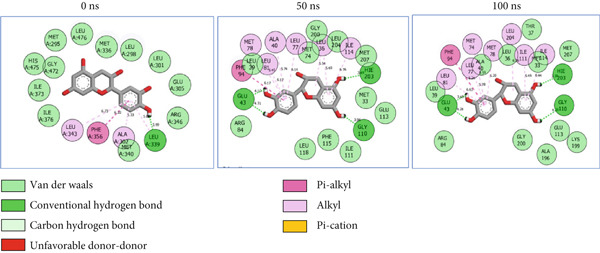
(c)

**Figure:**
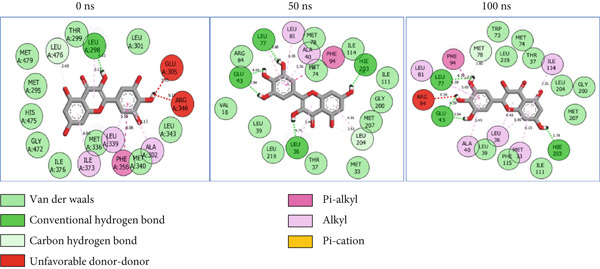
(d)

**Figure:**
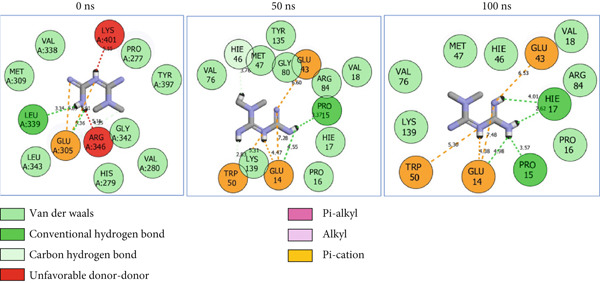
(e)

**Figure:**
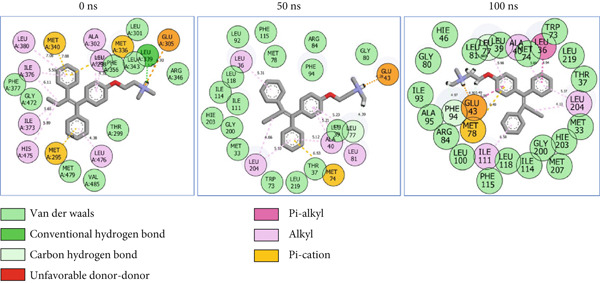
(f)

For ESR2, 21 interactions were observed between catechin and ESR2 at 0 ns with four H‐bonds (Ala302, Glu305, Arg346, and Gly472) while only 19 interacting bonds with two H‐bonds (Glu41 and Gly209) were observed at the end of the 100 ns. The ellagic–ESR2 complex had 19 interactions, including two H‐bonds (Gly472 and Thr299) at 0 ns, while 17 interactions with three H‐bonds (Hie212, Gly209, and Met76) existed at the 100 ns period (Figure 11b). There existed 16 interactions between epicatechin and ESR2 with one H‐bond (Leu339) at 0 ns and 22 interactions with three H‐bonds (Glu43, Hie203, and Gly110) after the 100 ns simulation period (Figure 11c). The myricetin–ESR2 complex formed 18 interactions with two H‐bonds (Leu298 and 476) at the 0 ns, while 21 interactions were observed at the 100 ns period with four H‐bonds (Met78, Leu77, Glu43, and Hie203) (Figure 11d). Metformin formed 12 interactions throughout the simulation period: one H‐bond (Leu339) and two H‐bonds (Hie17 and Pro15) with ESR2 at the 0 and 100 ns, respectively (Figure 10e), while tamoxifen formed 21 bonds (one H‐bond: Leu339) at 0 ns and 27 interactions with one H‐bond (Phe94) at the end of the simulation (Figure 10f).

## 4. Discussion

Medicinal plants contain a large reservoir of metabolites with therapeutic and nutritional values [41, 42] and are continuously explored for the development of improved antidiabetic drugs [43, 44]. The antidiabetic mechanism of action of *C. edulis* metabolites with focus on NP was investigated. Findings from this study revealed that 11 metabolites of *C. edulis* had H‐bond donors and acceptors < 10 and 5, respectively, with molecular weight ≤ 500 [45]; this implies that *C. edulis* contains metabolites with drug‐likeness potential. Genetic polymorphism identification of diseases helps in understanding the disease toward preventing the development of complications [46]. The 34 overlapping T2DM genes that interacted with 11 *C. edulis* metabolites in the PCT network suggest the association of *C. edulis* with T2DM.

The GO analyzes the functional processes underlying molecular mechanisms of metabolites and targets [47]. The observation that the negative regulation of apoptosis was the BP indicates that the metabolites could prevent and/or downregulate apoptotic processes and hence are capable of bringing about restoration of the functionality of the *β* cells as caused by the diabetic condition. Diabetes has also been reported to repress apoptotic regulatory genes, thus activating apoptotic pathways via a cascade of reactions that further leads to insulin resistance and mitochondrial dysfunction [48]. The antidiabetic activity of *C. edulis* was localized in the plasma membrane, and this probably connotes its ability to enhance the uptake of glucose via the GLUT 4 pathway to bring about normal blood glucose levels and make glucose available for utilization either for glycolysis, gluconeogenesis, or lipogenesis. Protein kinase acts in cells by downregulating different signaling pathways and targets, resulting in resistance to insulin, modulation of glucose tolerance, and inflammatory responses [49]. *C. edulis* metabolites probably act via the inhibition of protein kinase activity, thus shutting off or slowing down reactions that generate insulin resistance and improve glucose response and utilization.

The endocrine resistance pathway is implicated in resistance during endocrine therapy; therefore, it is necessary to explore the genes responsible for this resistance during the development of targeted drugs [50]. The estrogen receptor genes that were most enriched in the endocrine resistance pathway help in maintaining glucose homeostasis and energy metabolism by increasing insulin synthesis, sensitivity in hepatic cells, and preventing *β*‐cell apoptosis [51, 52]. Metabolites of *C. edulis* probably exhibit their antidiabetic activity by upregulating the ESR1 and ESR2 genes, resulting in enhanced cellular sensitivity to insulin via estrogen pathway activation. The other important genes in the endocrine resistance signaling pathway interacted with the *C. edulis* metabolites at varying levels. In T2DM, insulin‐stimulated AKT activation is reduced, leading to decreased insulin sensitivity and glucose disposal [53]. The EGFR gene is a cellular growth pathway regulator implicated in diabetic nephropathy [54] while IGF1R can activate cascades of intracellular proteins involved in glucose and lipid metabolism [55]. Matrix metalloproteinases (MMPs) are extracellular proteins of the matrix that degrade enzymes whose production might have been enhanced by high blood glucose, proinflammatory mediators, aldosterone, and oxidative stress [56]. The inhibition of SRC, a nonreceptor tyrosine kinase gene, results in improved production of insulin and decreased reactive oxygen species production [57]. The enrichment of these pathways in this study denotes the likely routes through which *C. edulis* metabolites could exert their antidiabetic effects.

NP is usually supported with MD simulation, as the latter helps to predict metabolites and targeted genes interaction for improved targeted drug development [58, 59]. The docking analysis provides insight into the ligand interaction with the target [60]. The observation that ellagic acid had the highest docking score with ESR1 but the least binding free energy suggests lesser binding affinity to the apo‐ESR1 than the other metabolites. The reduced binding free energy value could be due to the reduced number of interacting bonds formed during the simulation; this could have impacted its binding affinity for ESR1 relative to the other metabolites. However, catechin showed the highest binding affinity for ESR1, and epicatechin displayed the highest binding affinity for the ESR2 gene than the other *C. edulis* metabolites. Overall, *C. edulis* metabolites displayed greater binding affinity for the apo‐genes compared to the standard drugs, suggesting that the metabolites have greater antidiabetic impact than the standard drugs.

The degree of interaction relative to flexibility, stability, and active site amino acid residues is obtained from the MD study. The RMSD determines the extent of a complex’s stability by measuring the fluctuation pattern of complexes, with more converged complexes depicting stability [61]. The finding that catechin had a lower RMSD value than other metabolites and the standards indicates its superior binding stability and interaction, especially in H‐bond formation with the binding domain amino acids (Gly41, Gly209, and Hie212) of ESR1, which is important for modulating its activity. This is also evidenced by the highest binding affinity it exhibited for the gene. In ESR2, all the metabolites had lesser mean RMSD values compared to the standards, and the apo‐gene depicts better stability with ESR2. This is in line with the antidiabetic study of Akoonjee et al. [8], whereby lower mean RMSD values were noted for most of their profiled metabolites relative to their most enriched apo‐genes. Epicatechin and myricetin displayed higher docking scores and binding free affinities, which are reflected in their lower deviation from the apo‐ESR2. However, all complexes were < 3.5, with the metabolites converging with the genes better than the standards, which is in conformity with the high negative docking scores and binding free energies exhibited by the metabolites.

The RMSF can be used to determine the fluctuations occurring at the protein active site when a ligand/compound binds; the higher the flexibility of amino acids, the lower the stability of the complex [62]. The observed little or no significant peaks and lower RMSF values in the *C. edulis*‐ESR1 complex suggest less prominent vibrations and relative stability of the metabolites within the ESR1 binding domain amino acids, which are critical for its activity. The *C. edulis* metabolites–ESR2 complexes exhibited lower peaks compared to the standards and apo‐ESR2, with myricetin displaying the lowest mean RMSF value. This suggests that, unlike metformin and tamoxifen, the metabolites possess stable intermolecular interactions with the amino acid residues at the active site of ESR, as also evident by the high number of H‐bonds interaction at the end of the 100 ns simulation. The thermodynamic organization/conformation and folding of a ligand–receptor complex is measured by the RoG, where higher compactness and folding translate to complex stability and organization [63]. Except for protocatechuic acid‐3‐glucoside, the metabolites had lower RoG values relative to the apo‐ESR1, depicting fluctuation and hence less stable complex. However, this can be compensated for by the *Δ* *G*_bind_ values observed in all the metabolite–ESR1 complexes and the interaction at the active site of ESR1. The observation that *C. edulis* metabolites, except myricetin, had higher RoG values than the apo‐ESR2 and standards indicates greater compactness and stability with the apo‐ESR2. The extent to which a ligand–receptor complex has access to solvent can be determined by measuring the SASA [64]. The ESR1‐standard complexes displayed high SASA fluctuations throughout the simulation period, indicating lower thermodynamic stability with ESR1. This could have been influenced by their low docking scores and *Δ* *G*_bind_ values. However, *C. edulis* metabolites lower SASA values relative to the apo‐ESR1 are suggestive of thermodynamic stability with the ESR1. Epicatechin and myricetin–ESR2 complexes showed the least fluctuations relative to other metabolites and the standards; thus, both metabolites are presumed to be more thermodynamically stable than the other metabolites. This stability is probably influenced by their high *Δ* *G*_bind_ values.

The intermolecular interaction between ligand and protein depicts complex stability, and this can be predicted by the H‐bonds in the complex [65]. In this study, *C. edulis* metabolites had more H‐bond interactions than the standards, and this could have contributed to the stable complexes formed between *C. edulis* metabolites and ESR1. The 2D interaction plots of ESR1 and ESR2 with *C. edulis* metabolite post‐MD simulation showed varying types and numbers of interactions. H‐bond formation is important in a complex as it contributes to complex stability and lowers binding free energy for greater interaction [66]. An increase in the total number of interacting H‐bonds was observed between *C. edulis* metabolites and both apo‐genes after the 100 ns simulation. Protocatechuic acid‐3‐glucoside had the highest number of H‐bonds (four), followed by ellagic acid and epicatechin. However, ellagic acid might have presented a less stable complex due to its low *Δ* *G*_bind_ values. There was no H‐bond interaction between tamoxifen and ESR1, while metformin had two, which suggests lesser stability and structural interactions with ESR1. For ESR2, all the metabolites showed greater H‐bond interactions, with myricetin and epicatechin having four and three H‐bonds, respectively. Their stability, compactness, and structural organization are probably influenced by their *Δ* *G*_bind_ values observed in this study.

**Figure 12:**
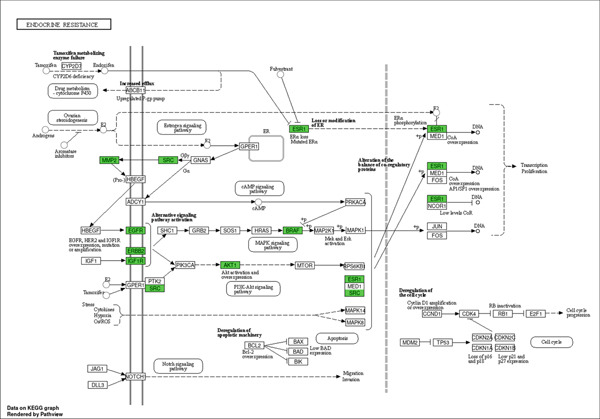
Enriched genes (green) in the endocrine resistance signaling pathway implicated with *C. edulis* metabolites as possible targets in the management of T2DM.

Insulin is an endocrine hormone responsible for the regulation of blood glucose [67]. T2DM is associated with a decrease in the transport of glucose by the glucose transporter pathway across the cell membrane, leading to reduced cellular glucose uptake [68]. Ellagic acid and epicatechin have been scientifically reported to be hypoglycemic metabolites that act by regulating the activity of alpha‐glucosidase, improving insulin secretion and sensitivity [69]. Myricetin improves glucose transport and uptake into the cells and acts as a glucagon‐like peptide‐1 agonist for insulin synthesis [70]. Protocatechuic acid enhances the release of insulin [71], while catechin improves insulin resistance and lowers blood glucose sources [72]. The findings that the endocrine resistance signaling pathway was the most significant and the estrogen receptors were the most enriched genes among the other identified genes (Figure 12) provide evidence for the possible antidiabetic mechanism of action of *C. edulis*.

## 5. Conclusion

The metabolites of *C. edulis* revealed drug‐likeness properties and antidiabetic potential by influencing insulin synthesis in the endocrine resistance signaling pathway. The interaction of *C. edulis* metabolites with T2DM genes probably results in the activation of proteins that enhance cellular glucose uptake via the activation of glucose receptors. Consequently, this study elucidates the probable anti‐T2DM molecular mechanism of action of *C. edulis* and the potential of *C. edulis* metabolites as antidiabetic drug candidates. However, further preclinical and clinical studies are required to substantiate *C. edulis* as an effective antidiabetic drug candidate.

## Conflicts of Interest

The authors declare no conflicts of interest.

## Author Contributions

Halimat Yusuf Lukman: investigation, formal analysis, data curation, writing—original draft preparation, and writing—reviewing and editing. Athika Rampadarath: data curation, formal analysis, methodology, and investigation. Stephen Amoo: supervision and writing—reviewing and editing. Saheed Sabiu: conceptualization, methodology, project administration, supervision, and writing—reviewing and editing.

## Funding

This study was funded by the South African Medical Research Council (SAMRC) (SRUG2204193723), the Technology Innovative Agency, and the National Research Foundation’s Competitive Programme for Rated Researchers Support (SRUG2204193723).

## Supporting information


**Supporting Information** Additional supporting information can be found online in the Supporting Information section. Table S1: Docking scores of *C. edulis* metabolites with ESR1 and ESR2 genes. Figure S1: Network construction of *C. edulis* metabolites with (a) EGFR, (b) MMP2, (c) MMP9, (d) IGFIR, (e) SRC, (f) ERBB2, (g) AKT1, and (h) BRAF. Figure S2: 2D docking interactions of (a) catechin, (b) ellagic acid, (c) epicatechin, (d) protocatechuic acid 3‐glucoside, (e) metformin, and (f) tamoxifen with ESR1. Figure S3: 2D docking interactions of (a) catechin, (b) ellagic acid, (c) epicatechin, (d) myricetin, (e) metformin, and (f) tamoxifen with ESR2.

## Data Availability

The data presented in this study are available in the article.
