# Supplementary material for: Mechanistic Exploration of Carpobrotus edulis Metabolites in Type 2 Diabetes Intervention Through Integrated Computational Approaches
Source: Biomed Res Int. 2025 Sep 24;2025:9170020. doi: 10.1155/bmri/9170020 (PMC12459310; doi:10.1155/bmri/9170020)
Supplement: Supplementary file 1 — Supporting Information Additional supporting information can be found online in the Supporting Information section. Table S1: Docking scores of C. edulis metabolites with ESR1 and ESR2 genes. Figure S1: Network construction of C. edulis metabolites with (a) EGFR, (b) MMP2, (c) MMP9, (d) IGFIR, (e) SRC, (f) ERBB2, (g) AKT1, and (h) BRAF. Figure S2: 2D docking interactions of (a) catechin, (b) ellagic acid, (c) epicatechin, (d) protocatechuic acid 3‐glucoside, (e) metformin, and (f) tamoxifen with ESR1. Figure S3: 2D docking interactions of (a) catechin, (b) ellagic acid, (c) epicatechin, (d) myricetin, (e) metformin, and (f) tamoxifen with ESR2. [file BMRI-2025-9170020-s001.docx]

**Table S1** Docking scores for the ESR1 and ESR2 genes interaction with *C. edulis* metabolites

| **Compound** | **Docking score (kcal/mol)** |
| --- | --- |
| **ESR1** |  |
| Protocatechuic acid 3-glucoside | -7.5 |
| Gallic acid | -5.7 |
| Caffeic acid | -6.7 |
| Sinapic acid | -6.6 |
| Ellagic acid | -9.4 |
| Catechin | -9.1 |
| Epicatechin | -8.7 |
| Tamoxifen | -6.8 |
| Metformin | -5.1 |
| **ESR2** |  |
| Ferulic acid | -6.5 |
| Gallic acid | -5.7 |
| Caffeic acid | -6.3 |
| Sinapic acid | -6.4 |
| Ellagic acid | -8.5 |
| Catechin | -8.5 |
| Epicatechin | -8.8 |
| Myricetin | -8.4 |
| Tamoxifen | -5.9 |
| Metformin | -5.1 |


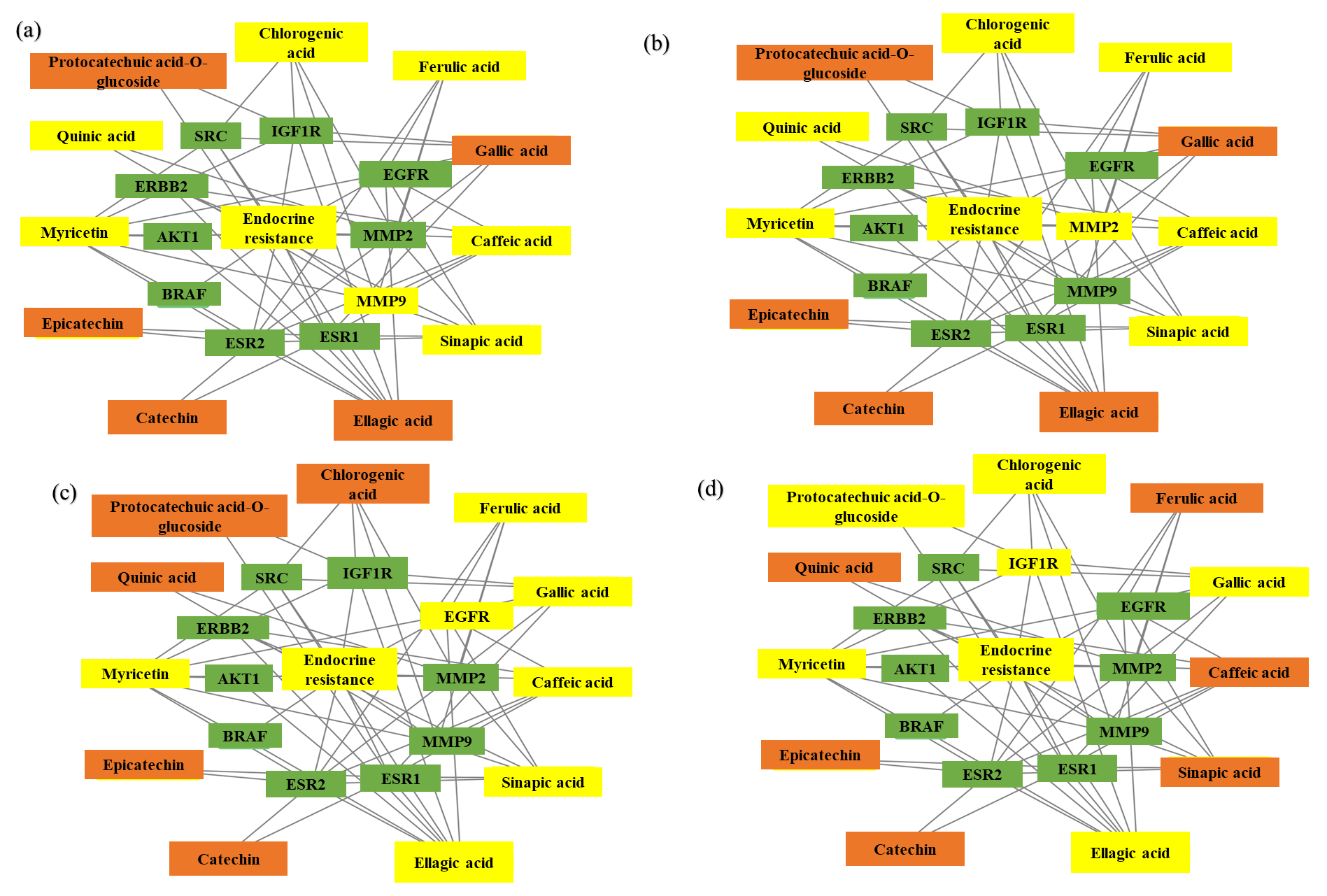


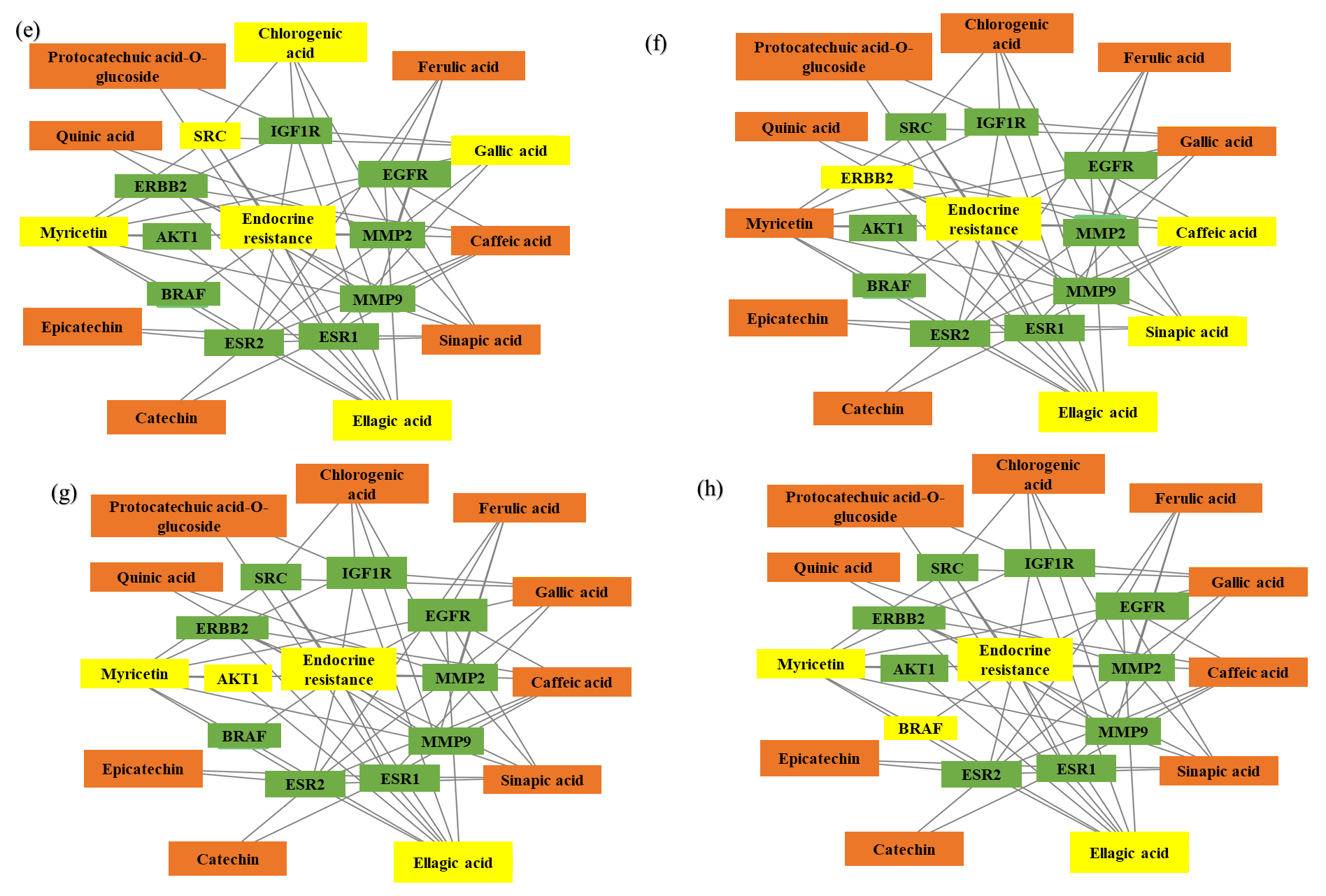


**Fig. S1** Network construction of *C. edulis* metabolites associated with other endocrine resistant genes. Interactions between *C. edulis* compounds (yellow) and endocrine resistance signalling pathway and genes (yellow) with (a) EGFR (b) MMP2 (c) MMP9 (d) IGFIR (e) SRC (f) ERBB2 (g) AKT1 (h) BRAF.


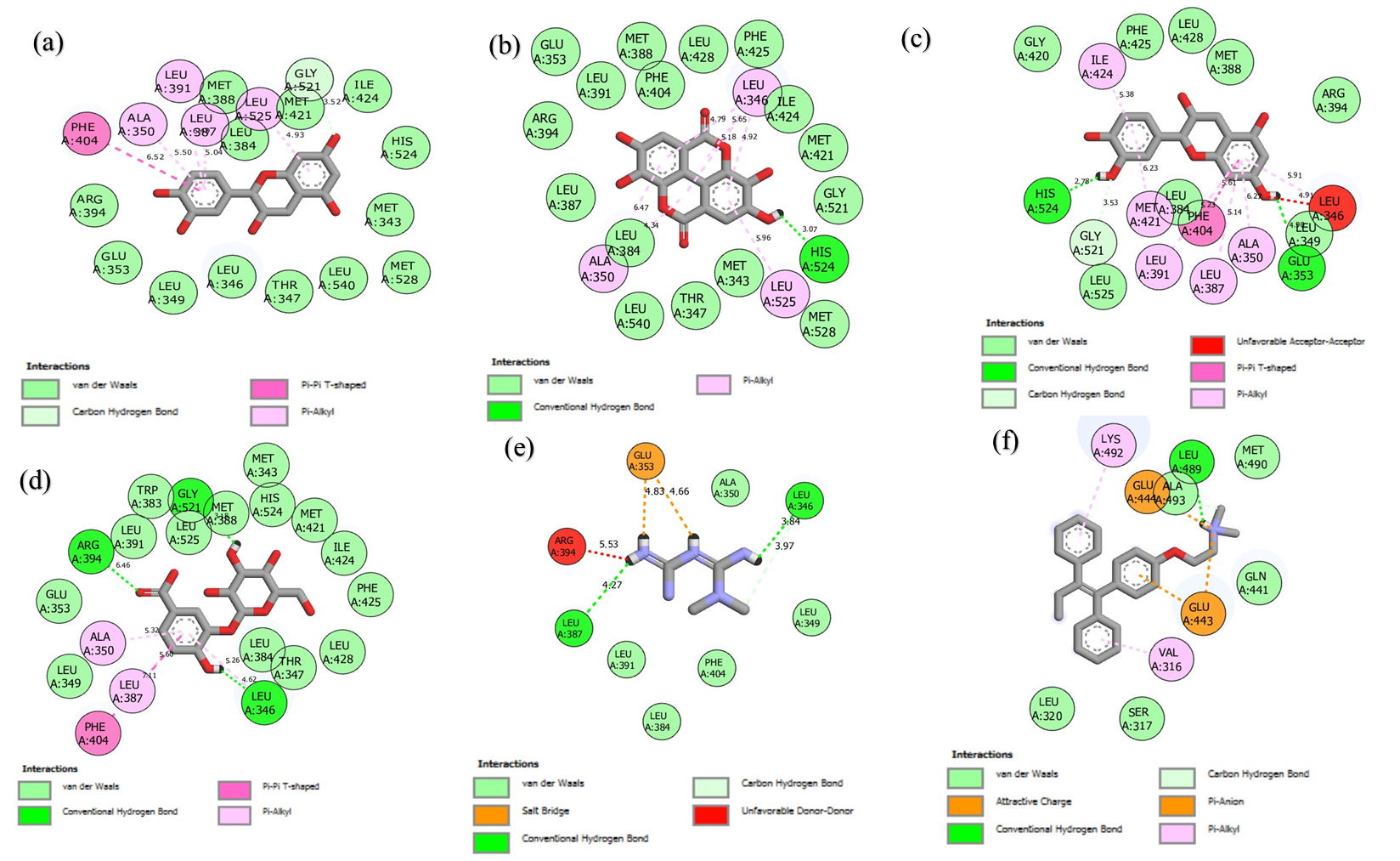
**Fig. S2** 2D Docking interactions of (a) catechin (b) ellagic acid (c) epicatechin (d) protocatechuic acid 3-glucoside (e) metformin, and (f) tamoxifen with ESR1.


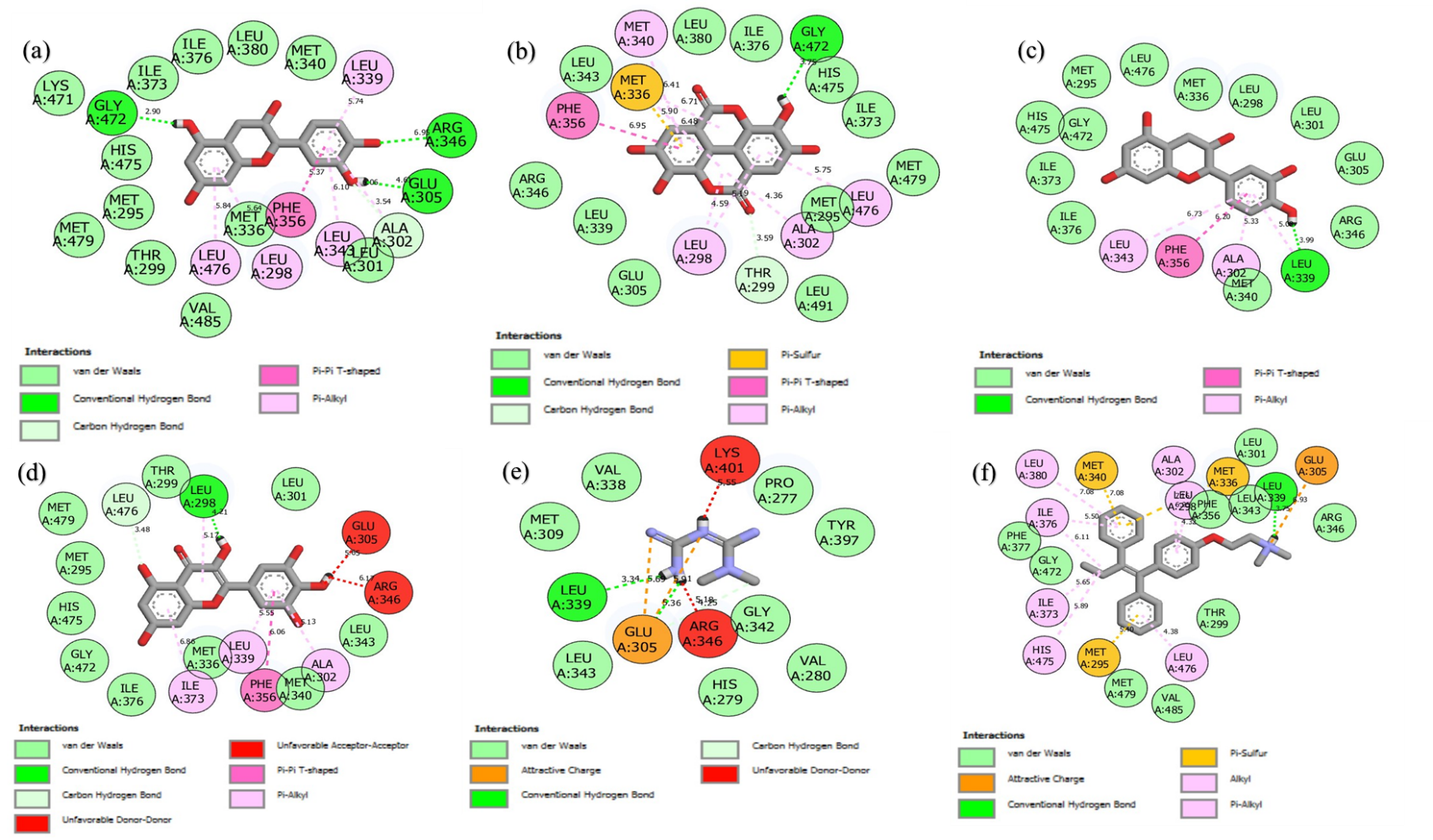
**Fig. S3** 2D Docking interactions of (a) catechin (b) ellagic acid (c) epicatechin (d) myricetin (e) metformin, and (f) tamoxifen with ESR2.
